# Erotic cue exposure increases neural reward responses without modulating temporal discounting

**DOI:** 10.1162/imag_a_00008

**Published:** 2023-08-18

**Authors:** Kilian Knauth, David Mathar, Bojana Kuzmanovic, Marc Tittgemeyer, Jan Peters

**Affiliations:** Department of Psychology, Biological Psychology, University of Cologne, Germany; Translational Neurocircuitry Group, Max Planck Institute for Metabolism Research Cologne, Cologne, Germany

**Keywords:** appetitive cue effects, temporal discounting, choice impulsivity, model-based fMRI

## Abstract

Humans prefer smaller sooner over larger later rewards, a tendency denoted as temporal discounting. Discounting of future rewards is increased in multiple maladaptive behaviors and clinical conditions. Although temporal discounting is stable over time, it is partly under contextual control. Appetitive (erotic) cues might increase preferences for immediate rewards, although evidence to date remains mixed. Reward circuit activity was hypothesized to drive increases in temporal discounting following cue exposure, yet this was never tested directly. We examined erotic vs. neutral cue exposure effects on subsequent temporal discounting in a preregistered within-subjects study in healthy male participants (n = 38). Functional magnetic resonance imaging assessed neural cue-reactivity, value-computations, and choice-related effects. We replicated previous findings of value-coding in ventromedial prefrontal cortices, striatum, and cingulate cortex. Likewise, as hypothesized, lateral prefrontal cortex activity increased during delayed reward choices, potentially reflecting cognitive control. Erotic cue exposure was associated with increased activity in attention and reward circuits. Contrary to preregistered hypotheses, temporal discounting was unaffected by cue exposure, and cue responses in reward circuits did not reliably predict changes in behavior. Our results raise doubts on the hypothesis that upregulation of (dopaminergic) reward systems following erotic cue exposure is sufficient to drive myopic approach behavior towards immediate rewards.

## 1 Introduction

People and many animals devalue future rewards as a function of time, resulting in an increased preference for immediate rewards (temporal discounting (TD); Kalenscher & Pennertz, 2008; Peters & Büchel, 2011). Despite high intra-individual stability (Bruder et al., 2021; Enkavi et al., 2019; Kirby, 2009), TD varies substantially across individuals (Peters & Büchel, 2011; Soman et al., 2005). High discount rates are observed in clinical groups exhibiting impulsive and/or short-sighted behavior (Bulley & Schacter, 2020), including gambling disorder, substance abuse, impulse control disorders, or lesions to the prefrontal cortices (Amlung et al., 2019; Garofalo et al., 2022; Lempert et al., 2019; Peters & D’Esposito, 2016; Weinsztok et al., 2021).

TD can be affected by environmental factors and cues (Lempert & Phelps, 2016; Peters & Büchel, 2011). In men, TD increases following block-wise presentation of arousing images of opposite-sex faces or erotica (Kim & Zauberman, 2013; Van den Bergh et al., 2007; Wilson & Daly, 2004), stimuli which possess inherently rewarding or appetitive qualities and elicit basic emotional responses (Klucken et al., 2013). More recent results support a more fine-graded association between visual appetitive stimulus processing and impulsivity, possibly moderated by internal motivational (e.g., mating mindset; see Chiou et al., 2015) or metabolic (e.g., hunger; see Otterbring & Sela, 2020) conditions (Chiou et al., 2015; Otterbring & Sela, 2020). Such internal states might foster active approach behavior towards immediate rewards.

Previous studies hypothesized that an upregulation of reward circuitry following appetitive cue exposure might drive this effect (Van den Bergh et al., 2007). Indeed, exposure to primary reinforcers including appetitive (erotic) cues increases activity in reward circuits, including ventral striatum (VS), orbitofrontal cortex (OFC), and ventral tegmental area (VTA; Brand, Snagowski, et al., 2016; Gola & Draps, 2018; Gola et al., 2016, 2017; Golec et al., 2021; Klein et al., 2020; Markert et al., 2021; Stark et al., 2019, 2022; Voon et al., 2014; Wehrum-Osinsky et al., 2014). Such exposure might also lead to a bias towards short-term rewards (Li, 2008; Mathar et al., 2022; Yeomans & Brace, 2015) possibly driven by increased dopamine (DA) release. Cortical and striatal dopamine tone have been shown to modulate TD (Arrondo et al., 2015; Cools, 2008; de Wit, 2002; Hamidovic et al., 2008; Kayser et al., 2012; Petzold et al., 2019; Pine et al., 2010; Wagner et al., 2020; Weber et al., 2016), although overall directionality of DA effects appears still mixed (D’Armour-Horvat & Leyton, 2014).

Erotic cue processing and a resulting present orientation in healthy participants might share conceptual similarities with cue-reactivity in addiction, referring to increased subjective, physiological, and neural responses to addiction-related cues (Courtney et al., 2015; Starcke et al., 2018; Volkow et al., 2010; Zhou et al., 2019). Exposure to gambling-related cues can drive increases in TD in gambling disorder (Dixon et al., 2006; Miedl et al., 2014; Wagner et al., 2022). Moreover, increased ventral striatal reactivity to erotic visual stimuli has been associated with the self-reported symptoms of Internet pornography addiction (Brand, Snagowski, et al., 2016), pornography use (Gola et al., 2017), and compulsive sexual behaviors (CSB; Gola & Draps, 2018; Voon et al., 2014).

The study of appetitive cue effects on TD in healthy participants might thus inform our understanding of maladaptive behaviors in clinical groups and potential interventions.

To sum up, there is considerable evidence that exposure to highly appetitive (erotic) cues can increase TD (Kim & Zauberman, 2013; Otterbring & Sela, 2020; Wilson & Daly, 2004) and that erotic cues upregulate activity in reward-related (dopaminergic) regions (Gola et al., 2016; Stark et al., 2005, 2019; Wehrum-Osinsky et al., 2014). However, the degree to which neuronal (erotic) cue-reactivity in these areas directly contributes to changes in TD remains unclear.

The current study addressed these issues in the following ways. First, extending previous work, we used fMRI to directly measure the effects of erotic cue exposure on reward circuit activity and subsequent temporal discounting. Second, we linked reward-system-reactivity to TD. Based on the previous literature, we preregistered the following hypotheses (https://osf.io/w5puk/):

- *Behavioral hypotheses*
  - H1: Temporal discounting will be selectively increased following erotic cue exposure. This effect will be driven by an enhanced bias towards smaller but sooner options
- *Neuronal hypotheses—replication of previous study findings*
  - H2: The subjective value (SV) of the delayed rewards (LL) will be coded in striatal and ventromedial prefrontal areas (vmPFC; see Peters & Büchel, 2009)
  - H3: Lateral prefrontal cortex activity (LPFC) will be increased during choices of LL vs. SS rewards (see Hare et al., 2014; Smith et al., 2018)
  - H4: Erotic vs. neutral cues will upregulate activity in a set of a priori-defined regions related to the processing of visual erotic stimuli (see Stark et al., 2019; a detailed procedure on ROI definition is outlined in the methods section)
- *Neuronal hypotheses—novel insights (linking neuronal cue effects to temporal discounting)*
  - H5: Lateral prefrontal cortex (lPFC) activity at onset of LL-option onset will be reduced following erotic vs. neutral cues
  - H6: Increased reward-system-reactivity (erotic>neutral) within key dopaminergic regions (Nacc, VTA) and reduced LPFC activity in response to erotic cues will both be positively associated with cue-induced increases in TD

## 2 Materials and Methods

### 2.1 Participants

Based on mean effect size estimates from two previous studies on erotic cue exposure effects on TD (Kim & Zauberman, 2013; Wilson & Daly, 2004), a power analysis (G*Power; Faul et al., 2007) yielded a preregistered sample size of N = 31 when taking a test-retest reliability estimate of TD into account (Enkavi et al., 2019) (effect size Cohen’s f = 0.22, error probability α = .05, power = .80; F-tests, number of groups: 1; number of measurements: 2; correlation between repeated measures: 0.65). To account for potential drop out and data loss, we tested a total sample of 38 participants. Two participants dropped out after the first testing session. fMRI data from one additional participant was lost due to technical error at the MRI environment, while behavioral data was preserved. The final sample therefore consisted of N = 36 male participants (mean age ± SD (range) = 31.2 ± 7.5 (20-50)). Participants were recruited via advertisements on Internet bulletin boards, mailing lists, and local notices. Main inclusion criteria included male gender, right-handedness, heterosexuality, normal or corrected-to-normal vision, no alcohol or drug abuse, no psychiatric, neurological, or cardiovascular disease (past or current), and no pacemakers or other ferromagnetic materials on the body. All experimental procedures were approved by the institutional ethics committee of the University of Cologne Medical Center (application number: 17-045), and participants provided informed written consent prior to participation in the study.

### 2.2 Appetitive cues

During each of the fMRI sessions, participants underwent two analogous cue exposure phases and performed two different decision-making tasks (see Tasks & Procedure section 2.3). Depending on the experimental condition of the day, participants were exposed to either erotic or neutral visual stimuli. Experimental images were partly derived from IAPS database, Nencki Affective Picture System (NAPS), EmoPics (Lang et al., 2008; Marchewka et al., 2014; Wessa et al., 2010) and from a google search. Our preliminary stimulus set consisted of 220 erotic and neutral images which were roughly matched for image content and complexity. In a preceding pilot study, the preliminary set was rated concerning valence and arousal levels by an independent sample. The most arousing erotic (N = 90) and the least arousing neutral images (N = 90) were included into our experimental image pool. Consequently, erotic and neutral cues differed in arousal (erotic: 65.07 ± 3.51, neutral: 4.89 ± 3.39; *t_(178)_* = 140.67, *p* < 0.001) and valence (erotic: 64.92 ± 3.39, neutral: 48.90 ± 9.84; *t_(178)_* = 14.59, *p* < 0.001). We ensured that images were matched on intensity (erotic: 0.46 ± 0.09, neutral: 0.45 ± 0.14; *t_(178)_* = 0.26, *p* = 0.79) and contrast (erotic: 0.19 ± 0.04, neutral: 0.19 ± 0.03; *t_(178)_* = -0.47, *p* = 0.64). Control scrambled images were created by randomly shuffling pixel locations, thereby preserving intensity and contrast. Unique image sets were created for each participant and for each cue phase by randomly drawing 40 intact and 20 scrambled control images without replacement from their respective image pools (N = 90).

### 2.3 Tasks & procedure

**Fig. 1.**
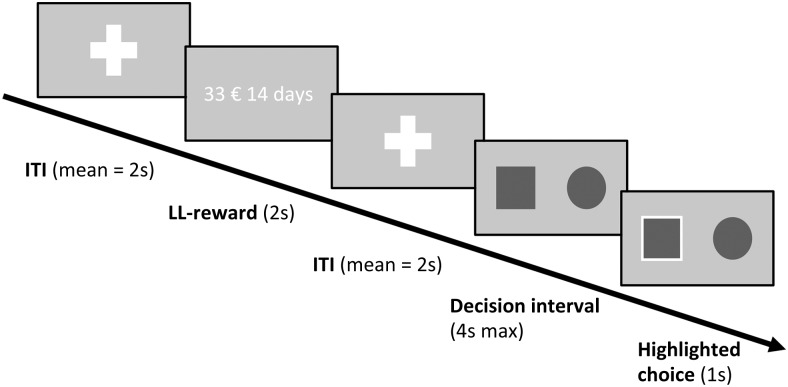
Example trial from the delay discounting task. Larger later reward (LL) presentation was preceded and followed by short jitter intervals (ITI), marked by white fixation crosses; Durations for the jitter intervals were sampled from a poisson distribution (M = 2 s; range: 1-9 s); Thereafter, the decision screen was presented. The fixed smaller sooner reward (SS; 20€) was never shown throughout the experiment.

The current study was conducted as one group within-subject design, including two experimental conditions (erotic vs. neutral). Data collection took place on two testing days with an approximate interval of 11 days (mean ± SD (range) = 11.31 ± 12.62 (1-70)). Each day, participants performed two decision-making tasks and two cue-exposure phases during fMRI. After introduction to the experimental set-up and scanning-preparation, participants completed the first cue-exposure phase. The cue phase consisted of 40 neutral or appetitive (erotic) images (depending on the condition on that day) and 20 scrambled control images which should be passively viewed. Each image was shown on the screen for a fixed duration of 6 s. To maintain participants’ attention, 10 trials were randomly chosen, in which participants had to indicate (via keypress) whether the last presented image depicted a person or not. We included an intertrial-interval (ITI) between consecutive image presentations, which was marked by a white fixation cross. The duration of the ITI was sampled from a poisson distribution (M = 2 s; range: 1-9 s). The total duration of the cue phase was 10 min. Following the first cue phase, participants completed 128 trials of a classical delay-discounting task (Peters & Büchel, 2009). On each trial, participants chose between a fixed immediate reward of 20€ (SS) and a variable delayed amount (LL). Every trial started with the presentation of the available LL-reward and the associated delay (e.g., 38€, 14 days). The LL-reward was depicted centrally on the screen for a fixed duration of 2 s. LL-presentation was followed by a short jitter interval which was marked by a white fixation cross. The jitter interval was included to better differentiate phases of valuation (LL-presentation phase) and choice for conducted fMRI analyses (see below). The duration of the jitter interval was sampled from a poisson distribution (M = 2 s; range: 1-9 s) and was followed by the decision screen. Here, participants chose between one of two symbols corresponding to the two options (SS: circle; LL: square). The response window was 4 s. The chosen option was highlighted for 1 s. The ITI was again marked by a white fixation cross with a presentation duration sampled from a poisson distribution (M = 2 s; range: 1-9 s). An example trial is depicted in Figure 1.

The LL-rewards were calculated beforehand by multiplying the SS-amount with two different sets of multipliers, differing slightly across days (Set 1: [1.01 1.02 1.05 1.10 1.15 1.25 1.35 1.45 1.65 1.85 2.05 2.25 2.65 3.05 3.45 3.85]; Set 2: [1.01 1.03 1.08 1.12 1.20 1.30 1.40 1.50 1.60 1.80 2.00 2.20 2.60 3.00 3.40 3.80]). We likewise used two sets of delays (Set 1: [1 3 5 8 14 30 60 122 days]; Set 2: [2 4 6 9 15 32 58 119 days]). Multiplier and delay combinations were randomly assigned to testing days per participant. Participants were instructed explicitly about the task structure and performed 10 test trials during a practice run within the scanner. In accordance with previous studies (Green et al., 1997; Mathar et al., 2022; Wagner et al., 2020), we used hypothetical choice options. However, note that discount rates for real and hypothetical rewards are highly correlated and similarly processed on the neuronal level (Bickel et al., 2009; Johnson & Bickel, 2002).

Following the TD task, participants underwent a second analogous cue phase, which was then followed by a reinforcement learning task (Two-Step task). This task is preregistered separately and will be reported elsewhere.

The second day followed exactly the same structure, with the exception of the cue phases. Depending on the condition on the first day, participants were presented with images from the other condition (neutral or erotic). The sequence was counterbalanced between participants (50% of the participants started with the erotic cue condition, and the other 50% were first presented with neutral cues). After completing the scanning session on the second day, participants performed three short working memory tasks (operation span (Foster et al., 2015), listening span (van den Noort et al., 2008), and digit span (Wechsler, 2008)) on a laptop and filled out a computerized questionnaire battery as well as a demographic survey. However, note that data from demographic, health, and personality questionnaires will be reported elsewhere.

### 2.4 Data analysis

#### 2.4.1 Behavioral data analysis of intertemporal choice

We used two different approaches to quantify impulsivity as measured by the TD task. Our model-based approach assumed hyperbolic devaluation of delayed rewards (Green & Myerson, 2004; Mazur, 1987) and a softmax choice rule for modeling subjects’ intertemporal decisions. For model-free analysis, we directly focused on actual choice preferences of SS- and LL-options.

##### Model-agnostic approach

A model-free analysis can avoid problems associated with the choice for a particular theoretical framework (e.g., hyperbolic discounting) or extreme parameter estimates that result in skewed distributions. The latter might yield problems for statistical approaches that require normally distributed variables. We therefore simply computed the relative proportion of SS-choices for every participant and condition (neutral vs. erotic) to obtain a model-agnostic measure of TD (Eq. 1).

$$TD_{model-agnostic}=\frac{Choices_{SS}}{\left(Choices_{SS}+Choices_{LL}\right)}$$ (1)

##### Computational modeling

We used hierarchical Bayesian modeling to fit a hyperbolic discounting model with softmax action selection to the choice data. This approach enables to separately assess cue condition effects on both steepness of temporal discounting and decision noise which cannot be disentangled via model-free approaches.

For each parameter (discount rate *k*, modeled in log-space, and inverse temperature *ß*), we fit separate group-level Gaussian distributions for the neutral condition from which individual subject parameters were drawn. To model condition effects on each parameter, we fit separate group-level distributions modeling deviations from the neutral condition for erotic cues, respectively (“shift”-parameter; Eqs. 2-3). Whereas higher *k*-parameters reflect an increased devaluation of the LL over time or more impulsive choice preferences, *ß* scales the influence of value differences on choice probabilities. Lower values of *ß* indicate a high choice stochasticity, whereas higher values indicate that choices depend more on value differences.

$$k_{\left(t\right)}=exp(k_{neut}+I_{Ero\left(t\right)}*S_{Ero_{k}})$$ (2)

$${ß}_{\left(t\right)}={ß}_{neut}+I_{Ero\left(t\right)}*S_{Ero_{ß}}$$ (3)

Here, *I_Ero_* is a dummy-coded indicator variable coding the experimental condition (1 = erotic, 0 = neutral) and *S_Ero_* are subject-specific parameters modeling changes in log(*k*) and *ß* depending on the condition in trial *t*. Computation of the discounted subjective value (*SV*) of the larger later option (*LL*) for a given delay *D* and amount *A* in a given trial then uses the standard hyperbolic model (Eq. 4):

$$SV_{\left(LL\right)}=\frac{\text{LL}}{\left(\mathrm{1+(}k_{\left(t\right)}*D)\right)}$$ (4)

However, cue exposure might also affect TD beyond a modulation of log(*k*), for example, by inducing an overall offset in the discounting function. To account for such effects, we examined another model that allowed for an offset in the discounting function in the neutral condition (modeled by the parameter $\omega_{Neut_{SV}}$), which might then again be differentially affected by erotic cues ($S_{Ero_{\omega }}$, Eq. 5).

$$SV_{\left(LL\right)}=SV_{\left(LL\right)}*(\omega_{neut}+I_{Ero\left(t\right)}*S_{Ero_{\omega }})$$ (5)

Because a positive $\omega_{Neut_{SV}}$ would indicate a subjective value of the LL that exceeds the objective amount (at delay = 0), the range of the offset-parameter was restricted between 0 and 1. Finally, subjective values of SS- and LL-options as well as modulated inverse temperature parameter *ß* (Eq. 3) were then used to calculate trial-wise choice probabilities according to a softmax choice rule:

$$P_{\left(chosen\right)}\;=\;\frac{exp(SV_{chosen}*{ß}_{\left(t\right)})}{exp(SV_{other}*{ß}_{\left(t\right)}) +exp(SV_{chosen}*{ß}_{\left(t\right)})}$$ (6)

In summary, we compared two models: Model 1 (*Base-model*) only included $S_{Ero_{\beta }}$ and $S_{Ero_{k}}$ to assess cue exposure effects on ß and log(k). Model 2 (*Offset-model*) additionally included a potential change in the offset, $S_{Ero_{\omega }}$.

###### Parameter estimation

Posterior parameter distributions were estimated via no-U-turn sampling (NUTS; Hoffmann & Gelman, 2014) implemented in STAN (Carpenter et al., 2017) using R (R Core Team, 2022) and the RStan Package (Stan Development Team, 2018). Prior distributions for the group-level parameters are listed in Table 1. Group mean priors were derived from posterior means and standard deviations from a recent study from our group, based on the Base-model (Mathar et al., 2022). STAN model code for all models is publicly available at OSF (Base-Model: osf.io/6uz8g; Offset-Model: osf.io/mgjx5). Model convergence was assessed via the Gelman-Rubinstein convergence diagnostic $\hat{R}$, and values of 1 ≤ $\hat{R}$ < 1.05 were considered acceptable. We ran 4 chains with a burn-in period of 1500 samples and no thinning. 4000 samples were then retained for further analysis. For details on MCMC convergence, see Gelman & Rubin (1992). We used Bayesian statistics (see Kruschke, 2010) to evaluate cue effects on model parameters of the best fitting model. Relative model fit was assessed via the loo-package in R using the Widely-Applicable Information Criterion (WAIC), where lower values reflect a superior fit of the model while considering its complexity (Vehtari et al., 2017; Watanabe, 2010).

**Table 1.** Priors of group-level parameter means

Base-model	Parameter	Group mean prior
	k	Normal (-4.2, 2.01)
	$S_{Ero_{k}}$	Normal (.15, .64)
	ß	Normal (.51, .3)
	$S_{Ero_{ß}}$	Normal (.02, .11)
Offset-model	Parameter	Group mean prior
	k	Normal (-4.2, 2.01)
	$S_{Ero_{k}}$	Normal (.15, .64)
	ß	Normal (.51, .3)
	$S_{Ero_{ß}}$	Normal (.02, .11)
	ω	Uniform (0, 1)
	$S_{Ero_{\omega }}$	Normal (0, .4)

We analyzed posterior distributions of group mean condition effects (as reflected in the *S*_Ero_ parameters, see Eqs. 2, 3, and 5 above) by computing their highest density intervals (HDI) and assessed their overlap with zero. We further report *undirected* Bayes factors (BF01) based on the Savage-Dickey-Density Ratio, which quantify the degree of evidence for a null model that would restrict a parameter of interest at a given value (e.g., *S_Ero_* = 0) against an alternative model where the parameter can vary freely (see Marsman & Wagenmakers, 2017 for details). To test the degree of evidence for increases vs. decreases in parameter values, we computed *directional* Bayes factors (dBFs) for condition effects. A dBF corresponds to the ratio of the posterior mass of the shift-parameter distribution below zero to the posterior mass above zero (Marsman & Wagenmakers, 2017). We considered Bayes Factors between 1 and 3 as anecdotal evidence, Bayes Factors above 3 as moderate evidence, and Bayes Factors above 10 as strong evidence. Likewise, the inverse of these values reflects evidence in favor of the opposite hypothesis (Beard et al., 2016).

##### Posterior predictive checks

We used posterior predictive checks to assess the degree to which the included models (Base-model, Offset-model) reproduced key patterns in the data, in particular the change in LL choice proportions across delays. For this purpose, we simulated 4000 datasets from each model’s posterior distribution and plotted the mean observed proportion of LL choices and the simulated LL choice proportions across delay. This was done separately for both conditions (neutral, erotic).

#### 2.4.2 fMRI data acquisition

MRI images were acquired on a 3 Tesla Magnetom Prisma Fit system (Siemens, Erlangen, Germany) equipped with a 64-channel head coil. Task stimuli were presented on an MR compatible screen and a rearview mirror system. Participants responded with their index and middle fingers on a two-button box, held in their right hand. Psychophysics Toolbox Version 3.52 implemented within MATLAB R2019b software (The Mathworks Inc., MA, USA) was used for stimulus presentation and behavioral data collection. Functional images were acquired in 5 separate runs (Cue phase1, TD, Cue phase 2, Two-step (first half), Two-step (second half)) by utilizing a multiband gradient echo-planar imaging (mb-EPI) sequence with repetition time (TR) = 0.7 s, echo time (TE) = 37 ms, flip angle = 52°, field of view (FOV) = 208 mm, voxel size = 2 mm³ isotropic (slice thickness = 2 mm, no gap), and multiband acceleration factor of 8. Each volume consisted of 72 transverse slices acquired in alternating order from the anterior-posterior commissure plane. The 5 runs contained ~7700 volumes for each participant and ~90 min of total scan time per day.

#### 2.4.3 fMRI data analysis

Preprocessing and analyses of fMRI data was performed using SPM12 (v7771; Wellcome Trust Centre for Neuroimaging) implemented in MATLAB R2019b (The MathWorks), and the FMRIB Software Library (FSL; Version 6.0.4; Jenkinson et al., 2012). Prior to statistical analysis, the first five functional volumes were discarded to allow for magnetic saturation. Functional images were corrected for motion and distortion artifacts using the FSL tools MCFLIRT and topup (Andersson et al., 2003; Smith et al., 2004). Next, anatomical T1-images were co-registered to functional images and normalized to the Montreal Neurological Institute (MNI) reference space using the unified segmentation approach in SPM12 (voxel size after normalization: [2,2,2] mm). Finally, we normalized functional images using the ensuing deformation parameters, and smoothed using a 6 mm full-width-half-maximum Gaussian kernel.

##### Cue phase

###### 1^st^/2^nd^ level modeling

Both testing days entailed two separate cue exposure phases (session 1 & 2). Note that to examine cue effects on TD, we only focused on the first cue exposure session directly preceding the TD task. In each cue phase, participants viewed 40 intact and 20 scrambled images. Depending on the condition of the day, intact images depicted either everyday scenes and portraits of people (neutral condition) or nude women (erotic condition).

Using SPM12, functional images from each day were analyzed using a general linear model (GLM). Each GLM examined the sustained activity during the presentation of intact and scrambled image types using boxcar regressors (duration = 6 s) which were convolved with the canonical hemodynamic response function (HRF). To account for residual variance caused by subject movement, we included the following nuisance regressors: 24 motion parameters (6 motion parameters relating to the current and the preceding volume, respectively, plus each of these matrices squared, see Friston et al., 1996), mean signal extracted from the ventricular cerebrospinal fluid (CSF), and a matrix containing motion-outlier volumes (identified by assessing global intensity differences between subsequent volumes; threshold: >75th percentile + 2.5 * interquartile range of the global signal).

Contrast images for intact and scrambled image presentation from the cue exposure phases (Cuephase1_Erotic;_ Cuephase1_Neutral_) were then entered into a second-level random effects model (flexible factorial design; factors: subjects, visibility (intact, scrambled), condition (erotic, neutral)) to evaluate BOLD-activity changes attributable to erotic image content. Variances for all factors were set to: *equal.* We included a main effect for “subject” and an interaction term for the factors “visibility” and “condition.”

We ran two analyses to evaluate neural effects of neutral vs. erotic cues. First, to replicate erotic cue effects (vs. intact neutral cues), we examined a priori-defined regions-of-interest (ROIs) related to the processing of visual erotic stimuli (see H4; Stark et al., 2019). The ROI mask was created using the group-level results (t-map) for the contrast erotic>neutral from Stark et al. (2019). For this purpose, we first used custom MATLAB code to filter out all voxels whose t-values fell below a cut-off of 6. Thereby we only kept the “most significant” voxels, showing increased responsiveness to erotic stimulus content. We then used small volume family wise error (FWE) correction (*p *< 0.05) across the entire mask to control for multiple comparisons. Further whole-brain effects of visual cue exposure are reported at an FWE-corrected threshold (*p* < 0.05; peak-level).

Second, we tested for associations between reward-system activity (erotic>neutral) within key dopaminergic (Nacc, VTA) and prefrontal (DLPFC) regions and behavioral cue effects on TD following erotic vs. neutral cue exposure (see H6). In more detail, we assessed associations between neuronal cue-reactivity-responses within the first cue phase (Erotic_session1_>Neutral_session1_) and subject-specific shift-parameters (S_Ero(k),_ S_Ero(ω)_), capturing condition-specific changes in TD. Associations were quantified via Bayesian correlations (using JASP (JASP Team, 2022; Version 0.14.3)) separately for predefined subcortical (Nacc, VTA) and cortical (DLPFC) ROIs.

To extract VTA activity, we first constructed an anatomical mask based on Stark and colleagues (2019; see above). Specifically, we used reported peak coordinates from the group contrast erotic>neutral (VTA: -6, -8, -10 and the mirrored location) as centers of two 10 mm spherical ROIs, which we then combined into a bilateral mask. For Nacc, we focused on the striatal cluster within the “reward” mask based on two meta-analyses, provided by the Rangel Neuroeconomics lab (http://www.rnl.caltech.edu/resources/index.html  ). This mask combines bilateral striatum, vmPFC, posterior cingulate cortex (PCC), and anterior cingulate cortex (ACC). Lastly for DLPFC, we built a custom mask based on previous studies reporting increased DLPFC-activity during LL vs. SS choices (Smith et al., 2018; see below). To calculate brain-behavior correlations, we first identified peak voxels from our group-level contrast erotic(intact)>neutral(intact) within the mentioned VTA, striatum, and DLPFC masks and extracted parameter estimates from these voxels for each participant.

##### Delay-discounting-task

###### 1^st^/2^nd^ level modeling

On both testing days, the first cue exposure phase was followed by a classical delay discounting task (see methods section 2.3). Functional images from both days (i.e., conditions) were analyzed separately using general linear models (GLM) implemented in SPM12. Each GLM included the following regressors: (1) the presentation of the larger later option (LL) as event regressor (duration = 2 s), standardized discounted subjective value (SV) as parametric modulator (computed based on the best-fitting model), (3) the onset of the decision period as stick regressor (duration = 0 s), and (4) the choice (LL vs. SS) as parametric modulator. Invalid trials on which the participant failed to respond within the response window (limit: 4 s) were modeled separately. All regressors were convolved with the canonical hemodynamic response function as provided by SPM12. Residual movement artifacts were corrected by using the same nuisance regressors as for the cue phase (see above).

We hypothesized subjective value (SV) of delayed rewards to be encoded in ventral striatal (VS) and ventromedial prefrontal areas (vmPFC) and that lateral prefrontal cortex activity (DLPFC) would be increased during choices of LL rewards (see H2 & H3). Further, we predicted that DLPFC activity during delayed reward presentation would be reduced following erotic cue exposure (see H5). To test H2 and H3, we entered the respective contrast images of parametric effects of subjective value (SV) and the chosen option (LL vs. SS) into separate second-level random effects models. We focused on predefined ROIs implicated in TD SV-computations (H2; Bartra et al., 2013; Clitero & Rangel, 2014) and choice behavior (H3; Smith et al., 2018). Specifically, H2 was tested using again the combined “reward” mask, which was provided by the Rangel Neuroeconomics lab (http://www.rnl.caltech.edu/resources/index.html), and combines bilateral striatum, vmPFC, PCC, and ACC. To test H3, we again used the above-mentioned DLPFC-mask created on findings on LL vs. SS choices (Smith et al., 2018). To control for multiple comparisons, we applied small volume correction (SVC; *p *< 0.05, peak-level) across the reward mask (H2) or the DLPFC mask (H3).

Finally, we tested for condition-related (erotic vs. neutral) differences in prefrontal activation related to the onset of the LL-rewards (H5). For this purpose, LL-onset regressors were directly compared between neutral and erotic image conditions on the group level. Here, we again used the above-mentioned preregistered DLPFC-ROI (Smith et al., 2018) for SVC (*p *< 0.05, peak-level).

#### 2.4.4 Deviations from preregistered analyses

This study was preregistered (https://osf.io/w5puk). We deviated from the preregistered analyses in the following ways: First, based on mean effect size estimates from two previous studies on erotic cue exposure effects on TD, we preregistered a minimum sample size of n = 31 to reach a power of .80 (effect size Cohen’s f = 0.22, error probability α = .05). To account for potential dropout, we aimed for a final sample size of n = 40. Due to technical issues of the MRI scanning environment, the final sample consisted of 38 subjects which was further reduced to 36, as two participants voluntarily dropped out of the experiment. Nevertheless, this still exceeds the minimum sample size by 5, indicating that we had enough power to detect potential erotic cue effects on TD.

Second, we slightly deviated from our planned computational modeling approach to quantify erotic cue effects on TD. We initially preregistered three models which all used hierarchical Bayesian modeling to fit variants of the hyperbolic model with softmax action selection to the choice data. However, two of the preregistered models suffered from two shortcomings (Model 2 & 3 in the preregistration). First, they both assumed cue-induced SV-offsets *only in the erotic condition*, thereby selectively increasing flexibility and predictive power in one condition. To correct this asymmetry, we now allowed for an offset of the discounting function in the neutral condition, which again could be differentially modulated by erotic cues (see Model 2, section 2.4.1). Second, the preregistered offset-parameter was initially defined as additive. However, validation analyses revealed that this formulation yielded implausible SVs (e.g., SV_LL_ < 0) in a few individuals who exhibited extremely unbalanced choice behavior (e.g., only very few SS or LL choices). Therefore, we changed the model formulation to a multiplicative offset (see Eq. 5).

## 3 Results

The results section is structured as follows. In accordance with our preregistered analysis plan, we first report the results of the replication analyses for the fMRI data for subjective value coding (H2), intertemporal choice (H3), and erotic cue processing (H4). Next, we report behavioral and modeling results regarding effects of cue exposure on TD (H1). Finally, to link neuronal cue-reactivity to TD, we report findings from two separate analyses. First, we assessed cue exposure effects on DLPFC activity at the during LL-reward presentation (H5). Second, we examined between-subjects associations between erotic reward-system-responsivity within key dopaminergic (Nacc, VTA) and prefrontal (DLPFC) areas, and alterations in TD (H6).

### 3.1 Neuronal correlates of subjective discounted value

We hypothesized subjective value (SV) coding of delayed rewards in striatal (VS) and ventromedial prefrontal areas (vmPFC; see Peters & Büchel, 2009; see H2). Our GLM incorporated the onset of the LL-option as event regressor (duration = 2 s) and the standardized discounted subjective value (SV) of the LL option as parametric modulator. SVs were based on the best-fitting *Offset-model* (methods section 2.4.2, lowest WAIC, see below). We used a combined “reward” ROI mask provided by the Rangel Neuroeconomics lab (http://www.rnl.caltech.edu/resources/index.html). This mask combines bilateral striatum, vmPFC, PCC, and ACC and was used for small-volume correction.

**Fig. 2.**
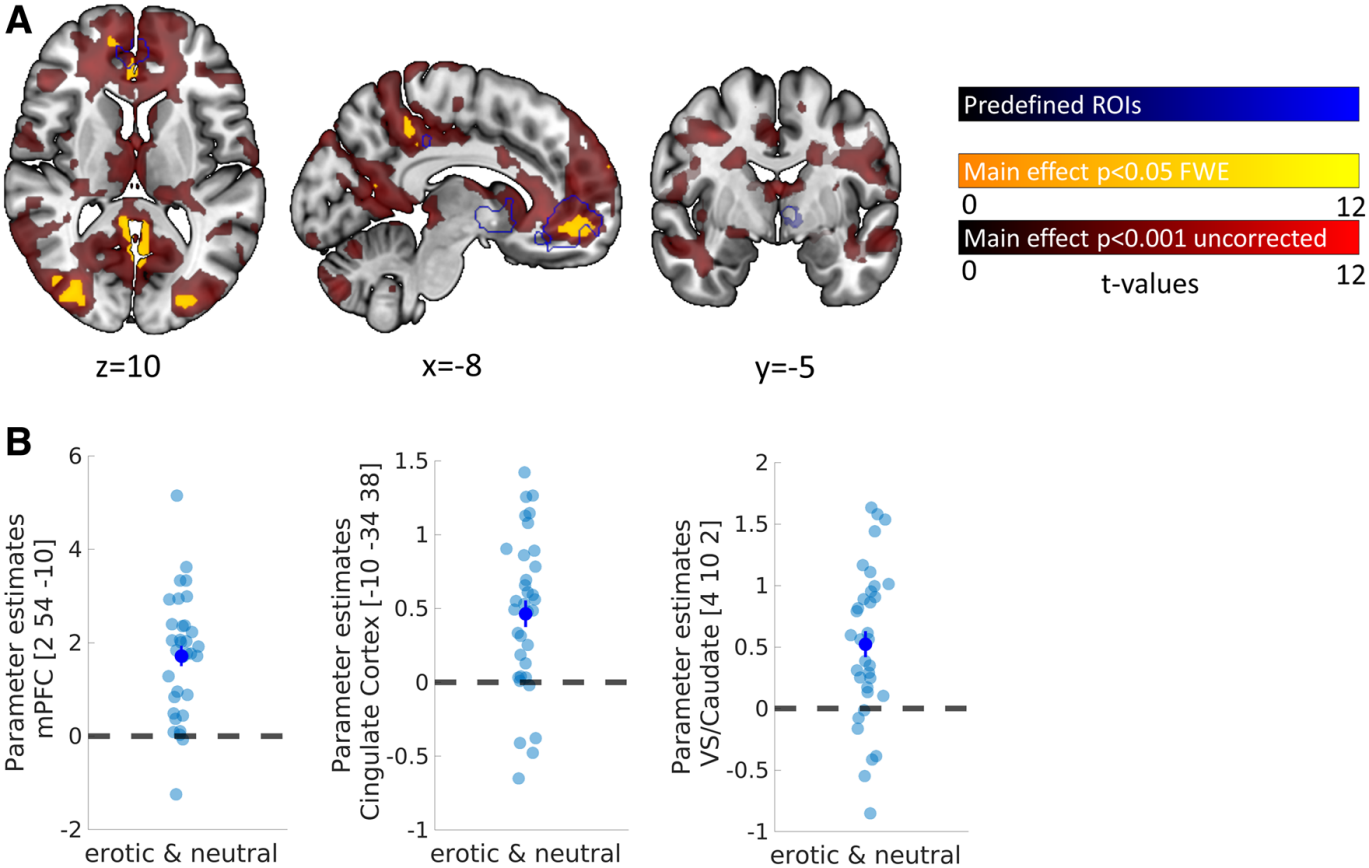
Neuronal correlates of subjective value (SV). (A) Display of the parametric SV-regressor (main effect across conditions); red, *p* < 0.001 (uncorrected); yellow, whole-brain FWE-corrected *p* < 0.05; blue, preregistered regions of interest from reward mask (see above); (B) Extracted *ß*-estimates of each participant extracted from medial prefrontal cortex (mPFC), posterior cingulate cortex (PCC), and ventral striatum/caudate (VS) peak coordinates of the parametric SV-regressor; error bars denote SEM.

Figure 2A shows brain activation that covaried with subjective discounted value of larger later rewards across experimental conditions (main effect across *erotic* and *neutral*). ROI analysis replicated previous findings on subjective value coding in a large cluster within medial prefrontal cortex (peak coordinates x*,* y*,* z (in mm): 2, 54, -10; z-value = 5.83, *p*_SVC_ < 0.001), posterior cingulate cortex (PCC; -10, -34, 38; z-value = 4.38, *p*_SVC_ = 0.005), and right ventral striatum/caudate (VS; 4, 10, 2; z-value = 4.22, *p*_SVC_ = 0.010)—confirming hypothesis H2. Parameter estimates extracted from vmPFC, PCC, and VS peak voxels illustrate that this effect was evident in the vast majority of individual participants (see Fig. 2B). Value-related activity in predefined ROIs did not differ between experimental conditions (no suprathreshold clusters for the contrasts: erotic>neutral or neutral>erotic). When running separate analyses for each condition, significant SV coding was confirmed in mPFC, PCC, and VS in the erotic condition (*p*_SVC_ < 0.05). In the neutral condition, this was true for the mPFC, VS reached trend level (see Supplementary Table S1). T-maps from the respective group-level contrasts are publicly available at OSF (https://osf.io/9uzm8/).

### 3.2 Neuronal correlates of intertemporal choice

We predicted increased dorsolateral prefrontal cortex activity (DLPFC) during choices of LL vs. SS rewards (Smith et al., 2018; see H3). Our GLM included the onset of the decision period as event regressor (duration = 0 s) and the selected option (LL vs. SS) as parametric modulator. We built (and preregistered) a custom (left) DLPFC-mask based using a 12 mm sphere centered at the group peak coordinates for the contrast “LL- vs. SS-choice” reported by Smith and colleagues (2018) (peak coordinates (x = -38, y = 38, z = 6)).

**Fig. 3.**
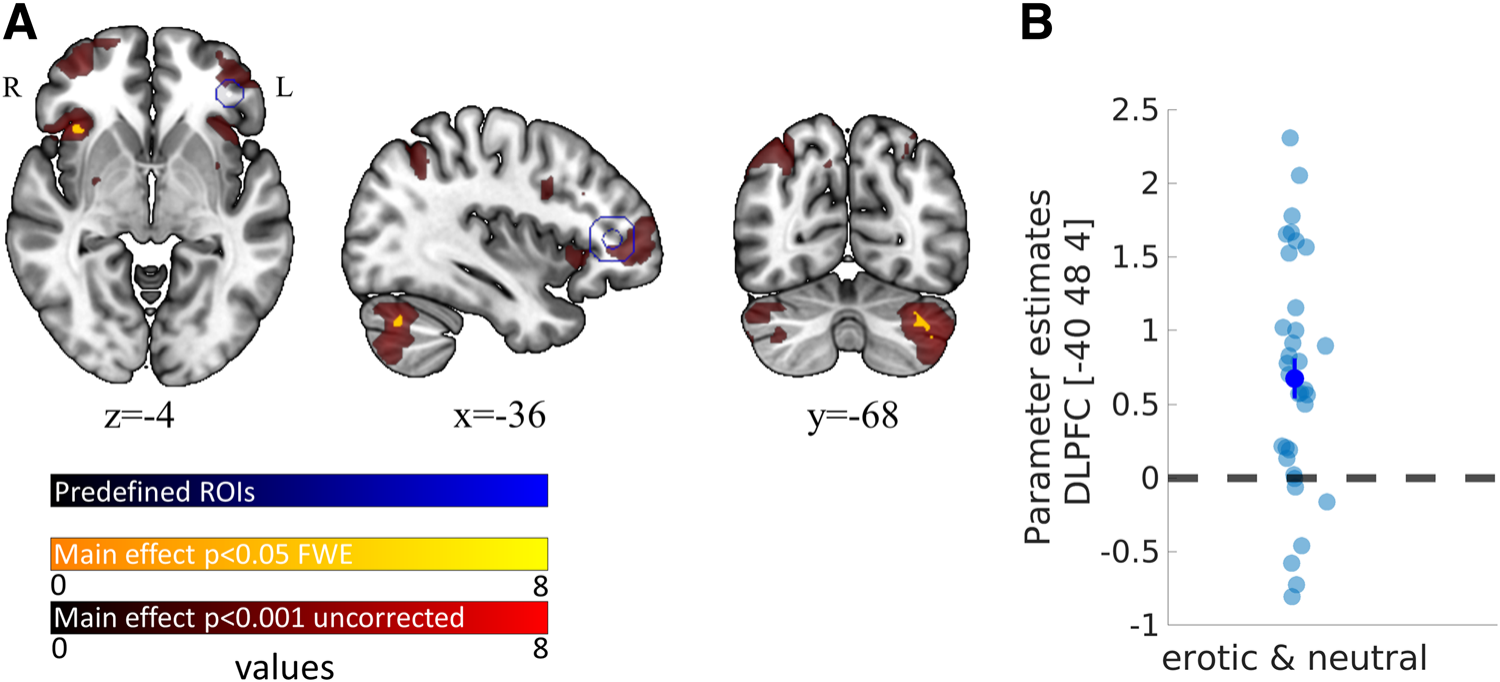
Neuronal correlates of larger-later (LL) vs. smaller-sooner (SS) choices. (A) LL>SS contrast (main effect across conditions); red, *p* < 0.001 (uncorrected); yellow, whole-brain FWE-corrected *p* < 0.05; blue, predefined regions of interest from custom DLPFC mask (see above); (B) *ß*-estimates of each participant extracted from left DLPFC peak coordinates; error bars denote SEM.

This ROI analysis replicated increased activity in left DLPFC associated with LL vs. SS choices across conditions (main effect across *erotic* and *neutral*; peak coordinates: -40, 48, 4; z-value = 4.26, *p*_SVC_ = 0.003; see Fig. 3), confirming hypothesis H3. We found no suprathreshold clusters for the contrasts: erotic>neutral or neutral>erotic. This effect was also confirmed in our preregistered ROI when each condition was analyzed separately (Supplementary Table S2). T-maps from the respective group-level contrasts are publicly available at OSF (https://osf.io/9uzm8/).

Subsequent whole-brain (FWE-corrected) analysis revealed two additional clusters coding for LL vs. SS choices across conditions (main effect across *erotic* and *neutral*), located in the right insular cortex (36, 20, -4; z-value = 5.38, *p*_FWE_ = 0.007) and the cerebellum (-34, 66, -34, z-value = 5.28, *p*_FWE_ = 0.012). We found no suprathreshold clusters for either condition contrast (erotic>neutral; neutral>erotic) using whole-brain FWE correction (*p *< 0.05).

In an exploratory whole-brain approach, we also checked for brain activity associated with choices of the immediately available option, that is the smaller but sooner option/reward (SS). Here, we found that brain activity within a multitude of cortical (cerebellum, mid-cingulate, bilateral insula, mid-frontal cortex) but especially subcortical regions (bilateral caudate, right putamen, thalamus, hippocampus) positively correlated with SS-choices across both experimental conditions (see Supplementary Fig. S1). For the condition contrasts, erotic>neutral and neutral>erotic however, no voxels survived whole-brain FWE correction (*p *< 0.05).

### 3.3 Appetitive cue effects on neuronal reward circuitry

We predicted (erotic-) cue effects on TD to be at least partly moderated by activations in neuronal reward circuits (Li, 2008; Stark et al., 2019; Yeomans & Brace, 2015). During the cue exposure phase, participants were exposed to 40 intact (erotic or neutral) and 20 scrambled control images. Analyses only focused on the first cue exposure session directly preceding the TD task. We ran a flexible factorial random-effects model (factors: visibility (intact/scrambled), condition (erotic/neutral)) and preregistered ROIs based on a previous study (Stark et al., 2019; see methods section for details). ROI analyses applied small-volume FWE correction (*p *< 0.05) across the entire mask.

A sanity check confirmed widespread functional responses across occipital and ventral temporal cortices for the intact vs. scrambled contrast (see Supplementary Fig. S2).

**Fig. 4.**
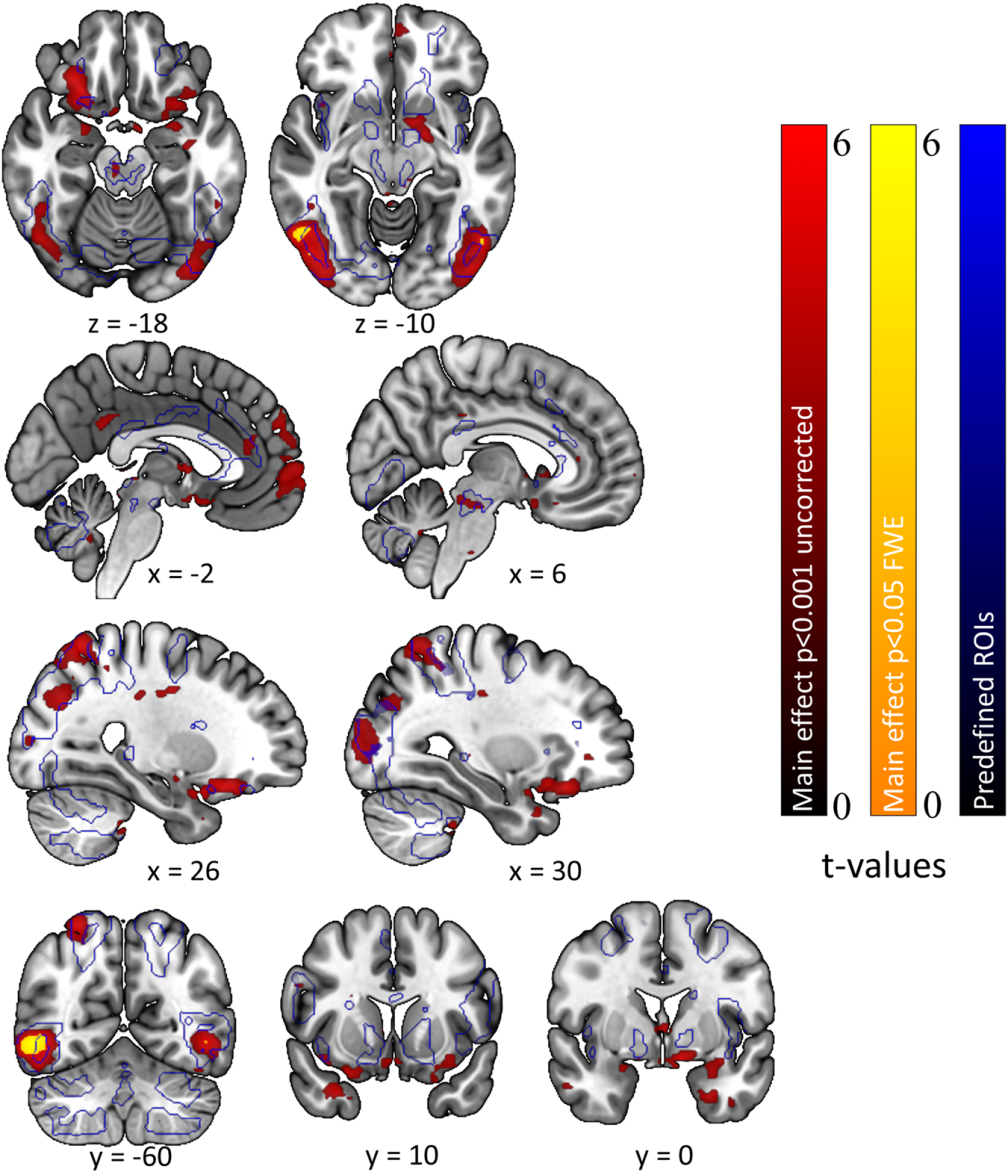
Neuronal correlates of (intact) experimental image processing (erotic>neutral). Red, *p* < 0.001 (uncorrected); yellow, whole-brain FWE-corrected *p* < 0.05; blue, predefined regions of interest from ROI mask (see above).

As depicted in Figure 4, (intact) erotic, compared to (intact) neutral cue exposure was associated with increased activity in widespread cortical and subcortical regions. Our preregistered ROI analysis revealed increased activity in four large posterior (cortical) clusters for erotic vs. neutral cues, including right inferior temporal cortex (52, -60, -4; z-value = 6.25, *p*_SVC_ < 0.001), left inferior occipital cortex (-48, -68, -6; z-value = 5.42, *p*_SVC_ = 0.001), right superior parietal cortex (26, -60, 62, z-value = 4.76, *p*_SVC_ = 0.013), and right middle occipital cortex (28, -72, 30; z-value = 4.51, *p*_SVC_ = 0.036).

**Fig. 5.**
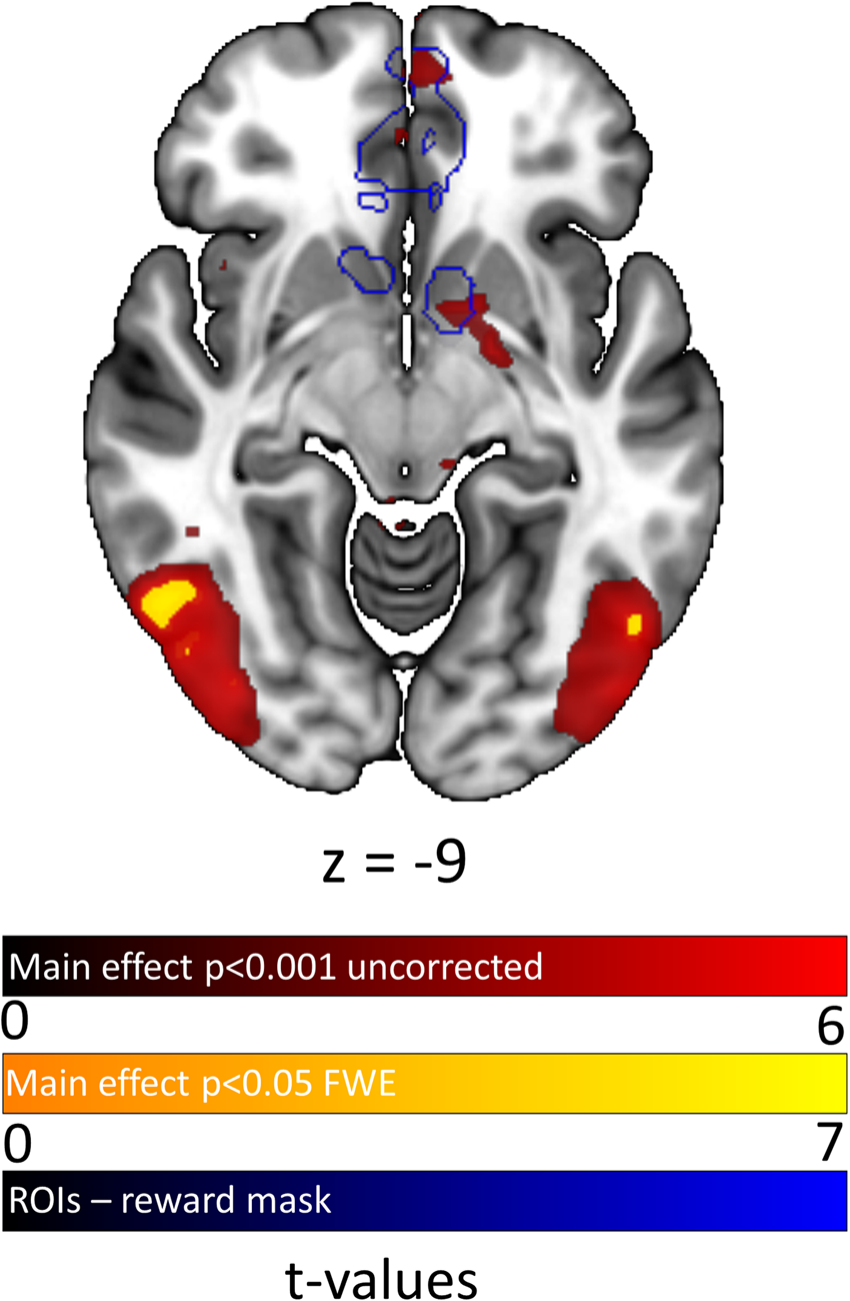
Neuronal correlates of (intact) experimental image processing (erotic>neutral). Red, *p* < 0.001 (uncorrected); yellow, whole-brain FWE-corrected *p* < 0.05; blue, regions of interest from reward mask (not preregistered, see above).

We had predicted subcortical activations in reward-related brain regions (e.g., VS, vmPFC) to be linked to erotic cue exposure (H4), but many subcortical effects fell just beyond the preregistered ROI-mask based on Stark et al. (2019). We therefore followed up with a second (not preregistered) ROI analysis using the above-mentioned “reward” mask, based on two meta-analyses, provided by the Rangel Neuroeconomics Lab (http://www.rnl.caltech.edu/resources/index.html). Small-volume correction was again applied across the entire mask. As expected, this confirmed highly robust bilateral effects in the VS/caudate (left: -10, 2, -10; z-value = 4.59; *p*_SVC_ = 0.002; right: 4, 6, 2; z-value = 3.70; *p*_SVC_ = 0.047) and the vmPFC (-6, 58, -2; z-value = 4.54; *p*_SVC_ = 0.002; see Figure 5).

A t-map depicting all activations associated with erotic>neutral image processing is publicly available at OSF (https://osf.io/9uzm8/). We also checked for increased brain activity following neutral compared to erotic image presentation. However, here we identified no suprathreshold clusters.

### 3.4 Appetitive cue effects on intertemporal choice

Having thus replicated previous findings on subjective value coding (H2), intertemporal choice (H3), and erotic stimulus processing (H4) (Peters & Büchel, 2009; Smith et al., 2018; Stark et al., 2019), we next assessed condition-related changes in TD behavior.

#### 3.4.1 Model-agnostic approach

**Fig. 6.**
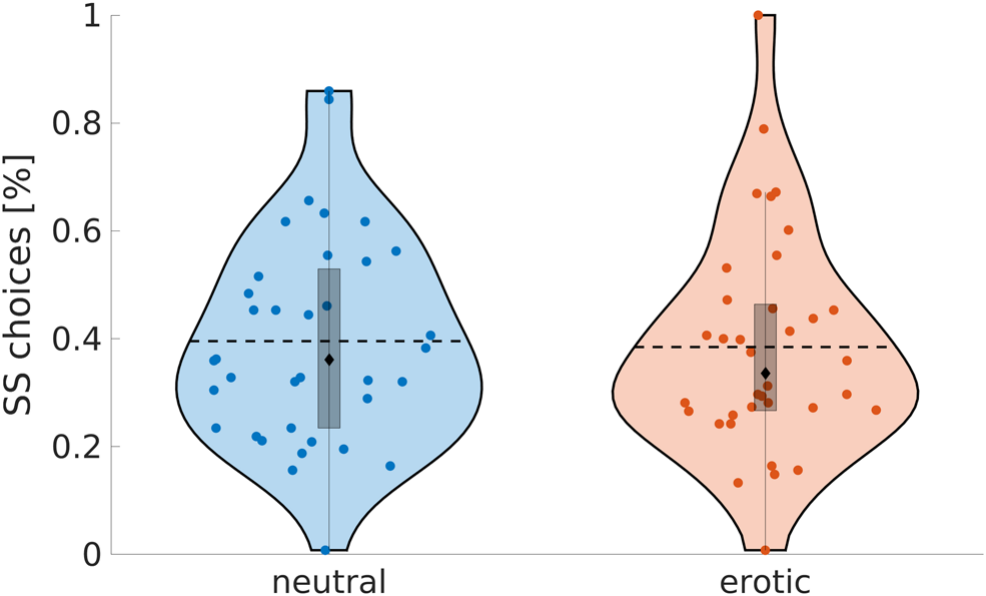
Percentage of smaller-sooner choices split by experimental condition (neutral vs. erotic). Colored dots = single subjects; Dashed lines = condition means; Black diamonds = condition medians; The edges of the boxes depict the 25th and 75th percentiles, and the whiskers extend to the most extreme datapoints the algorithm considers to be not outliers.

Contrary to our hypothesis (H1), TD was not differentially affected by appetitive cue exposure (see Fig. 6). In the neutral condition, the SS-option was chosen in 39.6% of trials whereas in the erotic condition the SS-option was chosen in 38.5% of trials (t_(35)_ = 0.714, *p* = 0.480).

#### 3.4.2 Computational modeling

**Fig. 7.**
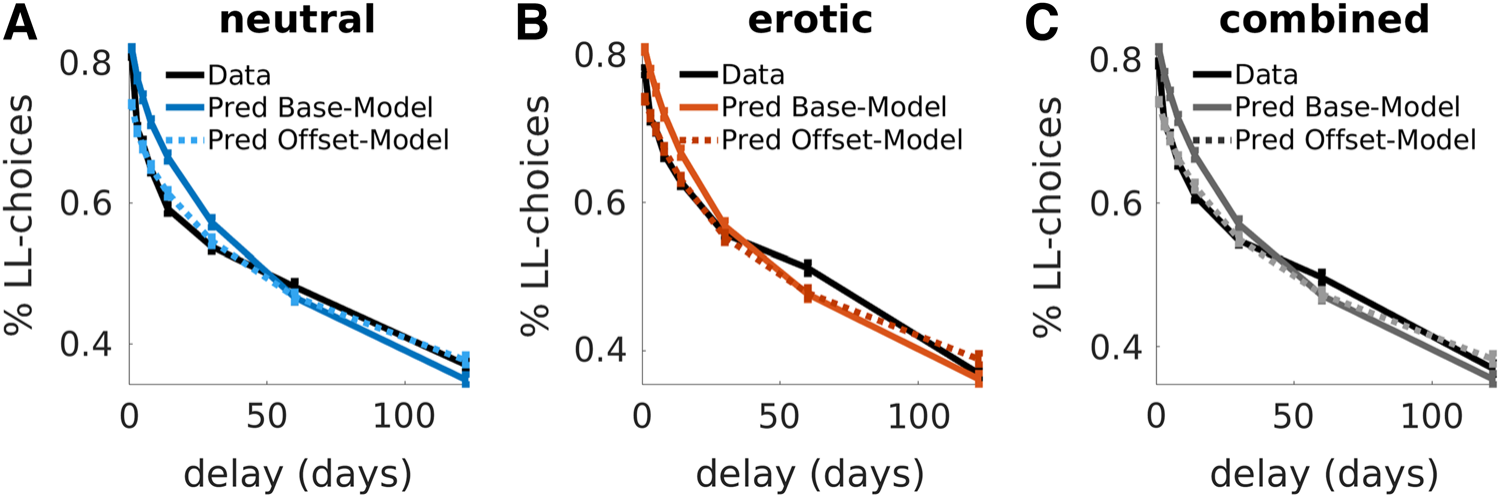
Group-level posterior predictive checks for the included temporal discounting models (Base-model, Offset-model). Here, we plotted the mean observed proportion of LL-choices and the simulated LL-choices from both models for each delay. Specifically, we created 4k simulated data sets from each model’s posterior distribution. For each simulated participant, we calculated the fraction of LL-choices across eight delay bins. Next, we calculated group average proportion of LL-choices for each delay and associated standard errors (vertical bars). Simulated data were then overlaid over the observed choice data. We did this separately for the neutral (A) and erotic (B) conditions as well as for the combined datasets (C).

Model comparison revealed that choice data were best captured by a hyperbolic model with an additional SV-offset-parameter *ω*, in addition to parameters accounting for choice consistency (*ß*) and steepness of TD (log(*k*); Offset-model). This model comparison replicated across conditions (neutral, erotic), and was confirmed in the combined model including parameters modeling condition effects (see Table 2). The superior fit of the offset-model was also reflected in choice predictions. The Offset-model accounted for around 82.2% (Base-model: 79.6%) of all decisions (Supplementary Table S3, Supplementary Fig. S3). Finally, posterior predictive checks confirmed that LL-choice proportions across delays were much better accounted for by the Offset-model (Fig. 7). All further analyses therefore focused on the Offset-model. However, note that due to an extreme behavioral choice pattern (only one single SS-choice in both conditions), data from one participant could not be explained by our winning model and was excluded from all further analyses.

**Table 2.** Summary of the WAICs of all included hyperbolic models in all sessions

Model	Neutral condition		Erotic condition		Combined	
	WAIC	Rank	WAIC	Rank	WAIC	Rank
Base-model	2654.537	2	2699.152	2	5365.867	2
Offset-model	2292.945	1	2453.293	1	4771.835	1

Note. Ranks are based on the lowest WAIC.

WAIC, Widely applicable information criterion.

**Fig. 8.**
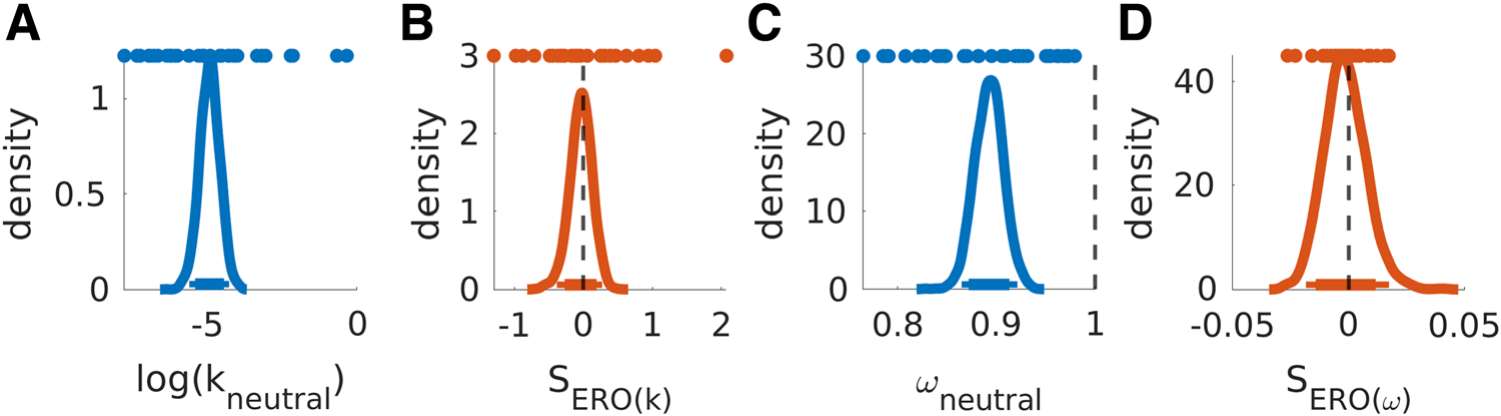
Posterior distributions for log(k_neutral_) and ω_neutral_ (A, C) as well as associated erotic shift parameters (SERO _(k, ω)_, B, D). Colored dots depict single-subject posterior means. Thick and thin horizontal lines indicate 85% and 95% highest density intervals.

**Fig. 9.**
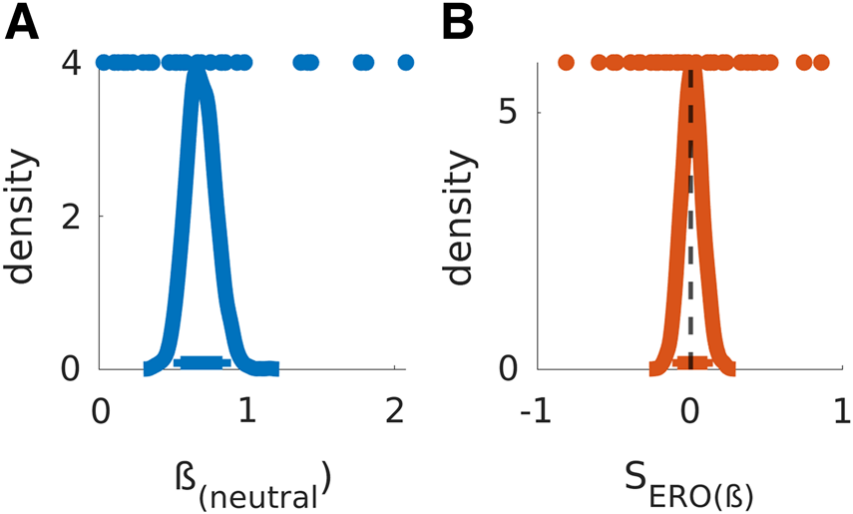
Posterior distributions for ß_neutral_ (A) and SERO_ß_ (B). Colored dots depict single-subject means. Thick and thin horizontal lines indicate 85% and 95% highest density intervals.

Examination of the posterior distributions of the best-fitting model then confirmed the model-agnostic results. TD (log(*k*); Fig. 8A) was not substantially affected by erotic cue exposure (SERO_(k)_; Fig. 8B), such that the highest density intervals for SERO_(k)_ substantially overlapped with zero. These data were more likely to be observed under a null hypothesis assuming SERO_(k)_ to be equal to zero (BF01 = 4.11). Interestingly, SV-offset parameters *ω*_neutral_ clearly differed from one in all participants, emphasizing the general utility of this additional parameter to account for a choice bias irrespective of delay. However, the observed data were much more compatible with the null model where the condition effect in the offset was equal to zero (BF01 = 43.18; Fig. 8C, D), strongly suggesting the offset was not modulated by erotic cue exposure. Likewise, data for the SERO_(_*_ß_*_)_ parameter were much more compatible with the null model, indicating that the change in stochasticity following erotic cue exposure was equal to zero (BF01 = 18.473, see Fig. 9A, B). See Table 3 for summary statistics and Bayes factors of the posterior distributions of all relevant parameters. For completeness, posterior distributions and Bayes factors from the inferior Base-model are reported in the supplement (Supplementary Fig. S4, Supplementary Table S4).

**Table 3.** Summary statistics of the posterior distributions of computational shift-parameters (offset-model)

Parameter	Mean	SD	dBF	BF_01_
SERO_(k)_	-0.050	0.634	1.450	4.113
SERO_(ω)_	-0.001	0.009	0.800	43.184
SERO_(ß)_	0.008	0.377	1.380	18.473

Note. BF_01_, undirected Bayes factor in favor of null model; dBF, directional Bayes factor; SD, standard deviation.

To confirm the validity of our modeling approach, we also examined associations between *SEro*_(_*_k_*_)_ and model-free measures of TD (SS-option choice proportions). Correlations between model parameters and model-free measures were consistently in the expected direction (see Supplementary Fig. S5, Supplementary materials).

### 3.5 Appetitive cue effects on neuronal and behavioral indices of temporal discounting

Despite increased (sub-) cortical processing of erotic compared to neutral cues, TD did not differ between experimental conditions. We next assessed the preregistered links between neuronal cue-reactivity and TD. We first report cue exposure effects on DLPFC activity during LL-reward presentation (H5), possibly indicating changes in (prefrontal) cognitive control. We next show between-subjects associations between erotic reward-system-responsivity within key dopaminergic (Nacc, VTA) and prefrontal (DLPFC) areas, and changes in TD (H6).

Recall that we reasoned (and preregistered) that cue effects on TD reported in previous studies (Kim & Zauberman, 2013; Van den Bergh et al., 2007; Wilson & Daly, 2004) might be due to cue-induced changes in prefrontal control regions and subcortical reward circuits. We tested the first prediction by comparing (left) DLPFC activity during LL-reward presentation (duration = 2 s) between experimental conditions (H5) using the preregistered DLPFC mask and small volume correction (12 mm sphere, peak coordinates (x = -38, y = 38, z = 6); Smith et al., 2018). Contrary to our hypothesis, we found no differences in DLPFC activity for the contrasts erotic> neutral or neutral>erotic. Likewise, on the whole-brain level no voxels survived FWE (*p *< 0.05) correction. A t-map depicting all activations associated with erotic> neutral LL-reward processing is publicly available at OSF (https://osf.io/9uzm8/).

Next, we tested associations between neuronal cue-reactivity-responses within key dopaminergic (Nacc, VTA) and prefrontal (DLPFC) areas and subject-specific condition effects on behavior (SERO_(k),_ SERO_(ω),_ H6), capturing individual differences of cue effects. Associations were quantified via Bayesian correlations (using JASP) separately for peak voxels from preregistered subcortical (Nacc, VTA) and cortical (DLPFC) ROIs (see methods section for details). We found no evidence for a significant correlation between functional cue-reactivity towards erotic cues and change in discounting behavior (SERO_(k),_ SERO_(ω)_). Contrarily, associations between cue-evoked changes in log(k) (SERO_(k)_) and subcortical ROI activity (Nacc, VTA) yielded highest BF01 (Nacc: 4.226; VTA: 4.663), indicating moderate evidence for a model assuming no association between dopaminergic brain activity and changes in *steepness* of TD. This model was approximately 4 to 4.5 times more likely than an alternative model given the data (see Table 4, upper panel; Supplementary Fig. S6, Supplementary materials).

However, we reasoned that characterizing cue-reactivity responses solely based on one peak-voxel could be problematic—potentially yielding biased estimates. Using mean voxel activity across the whole region of interest or respective sub-clusters might increase robustness (of approximations). In an exploratory approach, we therefore extracted average beta-values for the contrast erotic>neutral from above-mentioned VTA and DLPFC masks and the striatal cluster included in the reward mask. This analysis confirmed the non-significant association between SERO_(k)_ and brain activity across all three ROIs. Simultaneously, previous numerically negative correlation between SERO_(ω)_ and cue-reactivity within DLPFC and the ventral striatum (VS) was now even more pronounced, indicating that higher erotic cue-reactivity within these regions now appeared (significantly) associated with an increased preference for immediate (SS-) reward (see Table 4, lower panel; Supplementary Fig. S7, Supplementary materials). Although Bayes Factors (BF01) indicated at least moderate evidence for this association (DLPFC = 0.332: VS = 0.264), findings from this exploratory analysis should be interpreted with caution.

**Table 4.** Correlation statistics quantifying associations between brain activity in key dopaminergic (VS/Nacc, VTA) and prefrontal (DLPFC) areas and subject-specific shift-parameters (SERO(k), SERO (ω)) at the subject level

(A) Peak-voxel approach				
ROI peak voxel [x,y,z]	Model parameter	Correlation coefficient (r)	CI	BF01
DLPFC [-30, 36, 4]	SERO_(k)_	-0.259	[-0.531, 0.085]	1.638
	SERO_(ω)_	-0.239	[-0.516, 0.105]	1.932
VS/Nacc [-10, 2, -10]	SERO_(k)_	0.083	[-0.252, 0.393]	4.226
	SERO_(ω)_	-0.242	[-0.518, 0.102]	1.881
VTA [-10, 0, -12]	SERO_(k)_	-0.018	[-0.340, 0.309]	4.663
	SERO_(ω)_	-0.240	[-0.517, 0.104]	1.911

(B) Mean cluster activity approach				
ROI	Model parameter	Correlation coefficient (r)	CI	BF01
DLPFC	SERO_(k)_	-0.146	[-0.445, 0.194]	3.378
	SERO_(ω)_	-0.403	[-0.635, -0.068]	0.332
VS/Nacc	SERO_(k)_	0.061	[-0.272, 0.376]	4.432
	SERO_(ω)_	-0.416	[-0.644, -0.083]	0.264
VTA	SERO_(k)_	-0.117	[-0.422, 0.221]	3.801
	SERO_(ω)_	-0.218	[-0.501, 0.125]	2.237

Notes. ROI: Region of interest; CI: 95%-confidence interval; BF_01_, undirected Bayes factor in favor of null model; (A) Peak-Voxel approach: Beta-values were extracted from single peak-voxels within each ROI/sub-cluster; (B) Mean cluster activity approach: Average beta-values extracted from respective ROI/sub-cluster.

## 4 Discussion

Here, we followed up on the literature on erotic cue exposure effects on TD (Kim & Zauberman, 2013; Mathar et al., 2022; Van den Bergh et al., 2007; Wilson & Daly, 2004). We expanded previous work by leveraging a preregistered fMRI approach to assess cue exposure-related activity changes in prefrontal and subcortical reward-related brain areas, and by linking these effects to TD. We first replicated a range of effects from the imaging literature on TD, including subjective value coding in vmPFC, striatum, and cingulate cortex (Peters & Büchel, 2009), and increased left DLPFC activity for LL vs. SS choices (Smith et al., 2018). We also replicated the finding of increased visual and subcortical reward-related responses for erotic vs. neutral cues (Gola et al., 2016; Markert et al., 2021; Stark et al., 2019; Wehrum-Osinsky et al., 2014). However, these effects did not lead to increased TD, neither overall, nor in preregistered between-subject correlations focusing on key dopaminergic (Nacc, VTA) and prefrontal regions (DLPFC).

### 4.1 Neuronal correlates of subjective value and choice

We preregistered two replications for neural effects underlying TD. As predicted, and in line with previous work, activity in vmPFC, striatum, and cingulate cortex tracked subjective discounted value (SV) of LL-options (Bartra et al., 2013; Clitero & Rangel, 2014; Lee et al., 2021; Levy & Glimcher, 2012; Peters & Büchel, 2009; Sescousse et al., 2013). This effect was generally observed in most subjects and similarly evident following neutral and erotic cue exposure (at least for VMPFC and striatum). We found no evidence for condition differences in any of the reported clusters. This observation is inconsistent with the idea that upregulated activity levels, for example, in (dopaminergic) striatal regions following erotic cue exposure might disrupt subjective value encoding, which, in turn, might promote impulsive responding (Miedl et al., 2014).

We then focused on (left) dorsolateral prefrontal cortex (DLPFC), a region frequently implicated in TD (Guo & Feng, 2015; Hare et al., 2014) and self-control more generally (Hare et al., 2009). As preregistered, we observed increased decision-related left DLPFC activity for LL vs. SS choices. This pattern was observed across both experimental conditions (neutral, erotic), with no evidence for condition differences. Elevated DLPFC activity during LL choices (Smith et al., 2018) might be due to increased cognitive control during LL selections. This is supported by (1) increased TD following DLPFC disruption (Figner et al., 2010) and (2) fatigue effects manifested in increased TD that were associated with reduced DLPFC excitability (Blain et al., 2016). Our preregistered analyses therefore confirm an involvement of DLPFC, specifically in LL choices.

On the whole-brain level, two additional areas, right insular cortex and a cerebellar cluster showed increased activity for LL vs. SS choices. Whereas cerebellum has been observed in a wide range of tasks involving cognitive control and inhibition processes (Bellebaum & Daum, 2007; D’Mello et al., 2020; Stoodley & Schmahmann, 2009), insula activity was found to be specifically activated in LL-reward decisions and to depict a critical brain area involved in delaying gratification (Wittmann et al., 2007). This also resonates with findings from previous studies, reporting changes in insular activation in people with deficient foresight (Tsurumi et al., 2014), or reduced bilateral insula volumes in pathological gamblers compared with healthy controls (Mohammadi et al., 2016).

### 4.2 Appetitive cues affect neuronal reward circuitry

Exposure to appetitive visual cues, presented in a *blockwise* manner, can increase impulsive choice in subsequent TD tasks (Kim & Zauberman, 2013; Van den Bergh et al., 2007; Wilson & Daly, 2004). We reasoned such cue effects on TD to be at least in part driven by upregulated reward circuitry (Li, 2008; Stark et al., 2019; Yeomans & Brace, 2015), an account not directly tested before. We focused on predefined ROIs previously associated with erotic stimulus processing (Stark et al., 2019) and presented participants with 40 intact experimental (neutral, erotic) and 20 scrambled control images. A comparison of intact vs. scrambled visual image processing confirmed highly plausible activation patterns, including large clusters across occipital cortices and the entire visual stream (Margalit et al., 2017).

Exposure to (intact) erotic compared to (intact) neutral stimuli revealed increased activity in widespread cortical and subcortical brain areas. Preregistered ROI analysis (FWE_SVC_ < 0.05) yielded strong posterior occipital and temporal clusters showing increased cortical responses to erotic vs. neutral cues. However, subcortical effects in reward-related circuits (e.g., ventral striatum, vmPFC) in our data in many cases fell just beyond the ROI mask constructed from the Stark et al. (2019) data, which mainly contained more dorsal striatal effects. We therefore followed up with an additional ROI analysis that used the same reward mask that we used (and preregistered) for the subjective value analysis (bilateral striatum, vmPFC, PCC, and ACC) based on two meta-analyses (Bartra et al., 2013; Clitero & Rangel, 2014). This confirmed significant bilateral activations in ventral striatum and VMPFC.

Our results are consistent with previously reported erotic cue responses across stimulus types (images or videos) and sexes (Ferretti et al., 2005; Mitricheva et al., 2019; Stark et al., 2019). While effects in parietal and occipital cortices might reflect attentional orientation towards erotic vs. neutral stimuli, striatal and anterior cingulate effects might reflect the intrinsic value of erotic vs. neutral cues (Georgiadis & Kringelbach, 2012; Kuehn & Gallinat, 2011; Poeppl et al., 2016; Stark et al., 2019; Stoléru et al., 2012).

Neuronal cue-reactivity responses in visual regions largely overlapped with our preregistered ROI (based on group-level results (t-map) for the contrast erotic>neutral provided by Stark and colleagues (2019)). However, subcortical effects (e.g., in striatal regions) fell beyond the effects in the Stark et al. mask, and were instead located more ventrally, overlapping with the reward mask provided by the Rangel lab that we also used for the subjective value effects. We applied a binarization threshold (t-value = 6) to the entire T-map provided by Stark et al., to extract target voxels showing increased responsiveness to visual erotic stimuli. However Stark et al. (2019) used a somewhat longer stimulus duration (8 s vs. 6 s) and presented participants with both pictures and video clips to compare erotic vs. neutral cue reactivity responses. In their statistical analysis, they did not differentiate between both stimulus types to increase generalizability. Stark et al. also used an expectation/anticipation phase prior to image/video onset which cued the nature of the upcoming stimulus (erotic or neutral). These differences might have contributed to the somewhat more ventral striatal effects that we observed compared to Stark et al. (2019).

### 4.3 No evidence for temporal discounting changes following blockwise exposure to appetitive cues

We used model-free and model-based approaches to quantify TD. Whereas model-free analyses focused on raw choice proportions, our best-fitting computational model allowed us to separate cue effects on steepness of TD (log(k)) from a delay-independent offset in the discounting curve. H1 was not confirmed—TD measures were not differentially affected by erotic cue exposure. Instead, Bayesian statistics suggested moderate evidence for the null model. This contrasts with earlier findings from similarly design studies, reporting increased TD following blockwise exposure to erotic visual stimuli (Kim & Zauberman, 2013; Van den Bergh et al., 2007; Wilson & Daly, 2004). On the other hand, it is consistent with a recent study from our group (Mathar et al., 2022) that used a similar cue exposure design. In Mathar et al. (2022), we used psychophysiology rather than fMRI. The lack of jitter between trial phases thus allowed us to use comprehensive modeling of RT distributions using diffusion models. Cue exposure led to a robust change in the starting point of the diffusion process towards SS options, but, as in the present study, did not reliably affect log(*k*).

Multiple reasons could account for this discrepancy. First, we used fMRI to assess neuronal correlates of cue-exposure and TD. The scanning environment, including loud noises, narrowness, and movement restrictions, itself might have acted as an external stressor, possibly attenuating behavioral effects. Indeed, neuroendocrine stress parameters (salivary alpha amylase, cortisol) increase at the beginning of an fMRI session (Gosset et al., 2018; Lueken et al., 2012; Muehlhan et al., 2011), irrespective of stimulus presentation, and especially in scanner naïve participants (Tessner et al., 2006). Similarly, behavioral priming studies report smaller effects inside the scanner (Hommel et al., 2012), although such findings need replication. Both aspects might have contributed to an attenuation of behavioral cue effects in the current study. But, as noted above, in our earlier study (Mathar et al., 2022), cue exposure effects on log(k) were similarly largely absent, despite the lack of fMRI environment effects.

Further, our implementation of the cue-exposure phase differed slightly from previous approaches. Our cue phase was prolonged and included more experimental visual stimuli (n = 40) than earlier studies (max n = 25; Kim & Zauberman, 2013; Mathar et al., 2022; Van den Bergh et al., 2007; Wilson & Daly, 2004), although this should arguably have increased behavioral effects. We included additional design changes due to the fMRI design (scrambled control images, attention checks, jitter intervals between stimuli). These aspects could have attenuated the *continuous* blockwise character of cue-exposure, and concomitant rise in tonic dopaminergic tone, which might be required to affect TD (Pine et al., 2010). This resonates with previous studies showing that intermittent exposure to erotic cues is not sufficient to elevate TD (Knauth & Peters, 2022; Simmank et al., 2015).

Participants in our study passively viewed the presented images, rather than performing explicit arousal or valence ratings. However, explicit ratings might have induced deeper processing in earlier studies, which could have exhibited stronger effects on choice behavior (Van den Bergh et al., 2007; Wilson & Daly, 2004). Such attention effects can modulate behavioral (Gawronski et al., 2010) and neural effects (Anderson et al., 2003) of emotional stimuli. However, passive vs. active viewing of emotional images leads to similar physiological arousal effects (Snowden et al., 2016). Furthermore, our observation of increased activity in widespread cortical and subcortical networks in response to erotic vs. neutral control stimuli strongly argues against the idea that these cues were not adequately processed.

Although cue exposure was directly followed by the TD task, it could be argued that cue effects, and upregulated physiological reward circuit activity diminished rapidly over time, which might have also limited behavioral effects. However, we think two aspects speak against such idea. First, as already mentioned, our design largely mirrored previous experimental approaches which consistently detected cue effects on actual choice behavior (e.g., Wilson & Daly, 2004) or on more subtle bias parameters from computational models (Mathar et al., 2022). Moreover, a recent study from our lab (Knauth & Peters, 2022) also showed that trialwise emotional cue exposure (erotic, aversive, neutral visual cues) and associated upregulated arousal signals during the time of intertemporal choice were not sufficient to induce changes in TD.

Taken together, behavioral effects of erotic cue exposure on TD might not be as unequivocal as previously thought (Kim & Zauberman, 2013; Van den Bergh et al., 2007; Wilson & Daly, 2004). Recent studies utilizing trialwise erotic cue exposure failed to find changes in TD (Simmank et al., 2015). More critically, cue-evoked physiological arousal did not predict changes in discounting behavior (Knauth & Peters, 2022), casting doubt on the idea of an upregulated internal arousal state, that drives approach behavior towards immediate reward (Knauth & Peters, 2022). Also, recent *blockwise* studies question simple main effects of erotic cue exposure on impulsivity. Some studies find that cue exposure effects only occur under specific motivational or metabolic conditions (e.g., hunger; Otterbring & Sela, 2020). A noted above, we recently observed a robust change in the starting point of the evidence accumulation process towards SS rewards, which was revealed by extensive drift diffusion modeling of response time distributions (Mathar et al., 2022), whereas log(k) was largely unchanged. It is thus possible that the detection of cue exposure effects might require modeling of choices *and* response times. However, our fMRI-based experimental design separated option presentation responses, thereby precluding us from using comprehensive diffusion modeling of response times.

### 4.4 Elevated activity levels in dopaminergic brain areas cannot account for behavioral changes in temporal discounting

A major strength of the current study is its ability to empirically test the theoretical assumption of a cue-evoked upregulation in neural reward circuits, which might reflect increased dopaminergic activity (O’Sullivan et al., 2011; Redouté et al., 2000). Such effects might facilitate reward approach across domains (Van den Bergh et al., 2007). This idea is supported by pharmacological modulations of central dopamine transmission that affect TD (Arrondo et al., 2015; Cools, 2008; de Wit, 2002; Hamidovic et al., 2008; Kayser et al., 2012; Petzold et al., 2019; Pine et al., 2010; Wagner et al., 2020; Weber et al., 2016).

Here, we directly examined associations between neuronal cue-reactivity-responses towards erotic cues within key dopaminergic (Nacc, VTA) and prefrontal (DLPFC) areas and subject-specific condition effects on TD (SERO(k), SERO(ω)). However, if anything we found rather small evidence for our (preregistered) hypothesized association.

We first identified peak voxels from the group-level contrast erotic(intact)>neutral(intact) within all three above-mentioned ROIs and then correlated extracted beta-values with subject-specific shift parameters (SERO(ω), SERO(k)), as preregistered. This revealed no significant brain-behavior-associations. Based on feedback from a reviewer, we then ran an additional (exploratory) analysis, where we repeated above-mentioned analysis but now used average beta-values from the respective ROIs (DLPFC, VTA, ventral striatal sub-cluster within the preregistered reward mask). This confirmed non-significant association between SERO(k) and brain activity across ROIs. Further, we observed a small to moderate positive correlation between higher erotic cue-reactivity in VS and DLPFC and preference for immediate (SS-) rewards (corresponding to a more pronounced negative shift of the discounting curve offset). This association was numerically similar in the initial analysis, but now appeared more pronounced. However, these results should be cautiously interpreted for at least two reasons. First, while a positive correlation between myopic choice behavior and increased dopaminergic neurotransmission in the VS appears plausible, increased activity in DLPFC is harder to reconcile with this effect. DLPFC activity is often associated with cognitive control (Blain et al., 2016; Figner et al., 2010). Although cue-exposure phases did not entail any task requiring inhibition of prepotent impulsive responding, if anything, one would have expected decreased frontal activity to be related to SS-reward bias on the subject level. Further, the discrepancy between both approaches suggests that these brain-behavior correlations are susceptible to specific methodological details, which highlights that caution is warranted in their interpretation.

While a general dopaminergic impact on TD is well established, direction of reported effects in human studies appears somewhat inconsistent. Pine et al. (2010) observed increased TD following administration of the catecholamine precursor L-DOPA vs. placebo in a small sample of n = 14. In contrast, Petzold and colleagues (2019) observed no overall effect of L-DOPA administration on TD. Instead, effects depended on baseline impulsivity, supporting the view of an inverted-U-model of dopamine effects on cognitive control (Cools & D’Esposito, 2011). We recently observed (Wagner et al., 2020) reduced TD after a single low dose of the D2 receptor antagonist haloperidol, which is thought to increase striatal dopamine. The current study complements these previous findings and attempted to link (dopaminergic) reward system activity—which pharmacological approaches aim to evoke—to behavioral effects. However, upregulated reward system activity appears to be not sufficient to evoke *behavioral* cue effects (see previous section).

In the light of these contradictory findings, future studies should consider additional factors possibly involved in previously reported effects on TD. On the physiological level, arousal-related enhancement of noradrenaline (NE) release may be one possible mechanism (Ventura et al., 2008). Previous studies, indeed, found increased pupil dilation following highly arousing cues (Aston-Jones & Cohen, 2005; Finke et al., 2017; Kinner et al., 2017; Knauth & Peters, 2022; Murphy et al., 2014). NE agonists have been found to affect several forms of impulsivity (Robinson et al., 2008) and to directly increase the preference for LL rewards (Bizot et al., 2011). Further, Yohimbine, an α_2_-adrenergic receptor antagonist that increases NE release, reduced discounting in humans (Herman et al., 2019; Schippers et al., 2016). It appears highly plausible that (appetitive) cue-exposure will always affect both, noradrenergic and dopaminergic neurotransmitter systems.

### 4.5 Implications for addiction research

Appetitive cue effects on TD in healthy individuals might potentially also provide insights into mechanisms underlying maladaptive behaviors in clinical groups. Specifically, erotic cue effects on impulsive choice may partly resemble cue-reactivity processes in addiction. Drug cues trigger increased subjective, physiological, and neural responses which are associated with increased cravings, impulsive choice, and higher relapse rates (Preston et al., 2018; Vafaie & Kober, 2022). We initially hypothesized two potential routes through which erotic cues could have impacted TD. First, cue exposure could have interfered with (sub-) cortical value coding, thereby diminishing subjective perception of objective reward differences, promoting SS-option preferences. Similar findings have been reported in gambling disorder, when highly arousing gambling cues were presented (Miedl et al., 2014). Second, erotic cues could have impaired executive (cognitive) control over short-sighted choice behavior. Models such as the Interaction of Person-Affect-Cognition-Execution (I-PACE; Brand, Young, et al., 2016) model suggest an imbalance between executive control and reward networks in addicted individuals, which may be further exacerbated by cue exposure and contribute to disadvantageous decision-making. Our findings contribute to these considerations by demonstrating largely unaltered value coding and largely intact prefrontal executive control following exposure to non-drug-related erotic cues.

Notably, the analogy between erotic (appetitive) cue effects in healthy participants and addiction-related cue effects in addiction is complicated by several potential differences. Specifically, evoked cue-reactivity in the two cases might differ both quantitatively and qualitatively. While erotic cues in healthy participants might signal the upcoming occurrence of a pleasurable stimulus (learned via positive reinforcement), addiction-related cues might act via both positive *and* negative reinforcement routes. Over the course of addiction, cue exposure might be associated not only with rewarding (mesolimbic) effects but also with reductions of subjective craving and withdrawal symptoms. Similarly, recent evidence on the development of addiction-like, pathological use of sexual erotic material (SEM) also suggested that escalated impulsive or addictive behavior towards sexual material (compared to recreational use) might be fostered by both, negative and positive reinforcement processes (Brand et al., 2019; Brand, 2022; Stark et al., 2022). Resonating with this idea, Stark et al. (2022) found that elevated stress (indicated by cortisol responses) enhanced the neural reward activation to erotic material, suggesting that the behavioral relevance of reward cues might be strongly affected by the specific expectation (e.g., pleasure vs. stress reduction). Such expectation effects likely differ substantially between healthy subjects and those suffering from addiction. Future studies on erotic cue effects might therefore assess the motivation for the use of erotic stimulus material, as this might moderate potential cue effects and might highlight driving factors of a dysfunctional cue-reactivity response.

### 4.6 Limitations

Our study has a few limitations that need to be acknowledged. First, we only tested male heterosexual participants. Men and women might differ in neuronal responses to affective stimulus material and emotional processing (Bradley et al., 2001; Lithari et al., 2010; Wrase et al., 2003), although a recent meta-analysis found at most negligible sex differences in neural correlates of sexual arousal (Mitricheva et al., 2019). However, to extend generalizability of results, future studies should include participants from both sexes and different sexual orientations.

Second, we did not include an image rating task, capturing arousal, valence, or related dimensions. Therefore, we cannot directly quantify subjective arousal-associated individual cues. However, fMRI revealed substantial differences in neural responses to erotic vs. neutral cues in plausible brain regions implicated in attention and reward. Further, a pilot study in an independent sample confirmed that the applied stimulus material clearly modulated subjective arousal. Still, future studies might complement fMRI and task-based measures with self-reported arousal.

Third, we did not include an additional aversive cue condition to control for unspecific arousal effects. Previously reported erotic cue effects on TD might be at least partly attributable to increased arousal, although aversive cue effects on TD likewise appear mixed (Cai et al., 2019; Guan et al., 2015; Knauth & Peters, 2022). Nonetheless, it would be interesting to assess whether neuronal measures of aversive cue processing are predictive for choices.

Lastly, on each trial, participants only viewed the LL option, whereas the SS reward was fixed and never shown on the screen, as done in numerous earlier studies (Kable & Glimcher, 2007; Peters & Büchel, 2009). However, additionally displaying the smaller sooner reward (separated by a further jitter interval) could be interesting for two reasons. First, although we showed that value computations for LL rewards were largely unaffected by cue condition, neuronal representations of an *immediate* reward might have been affected by cue condition. Second, elevated dopamine tone might foster approach behavior towards rewards that appear spatially near or available. Only presenting one of two possible choice options instead of both (Guan et al., 2015) might have biased or even compensated cue effects.

### 4.7 Conclusion

Previous studies indicated that highly appetitive stimuli might increase TD behavior (Kim & Zauberman, 2013; Otterbring & Sela, 2020; Wilson & Daly, 2004). Cue-reactivity in reward-related circuits was suspected as a potential mechanism underlying these effects (Van den Bergh et al., 2007). Here, we leveraged combined fMRI during both cue exposure and decision-making to link activity in reward circuits to changes in TD. We first replicated core neural effects underlying TD (value coding in vmPFC, striatum, and posterior cingulate, LL-choice-related activity in DLPFC) (Bartra et al., 2013; Clitero & Rangel, 2014; Kable & Glimcher, 2007; Peters & Büchel, 2009; Smith et al., 2018). Further, we confirmed increased (sub-) cortical processing during erotic vs. neutral cue exposure in core regions of the reward circuit. However, our preregistered hypothesis of increased TD following erotic cue exposure was not confirmed. This resonates with recent findings from our lab, where such effects were only observed for the bias parameter in the drift diffusion model, and not for choice behavior per se (Mathar et al., 2022). Importantly, and in contrast to our preregistered hypothesis, activity in key reward regions (Nacc, VTA) did not predict changes in behavior. Our results cast doubt on the hypothesis that upregulated activity in the reward system is sufficient to drive myopic approach behavior towards immediately available rewards.

## Data and Code Availability

T-maps of 2nd-level contrasts as well as STAN model code and raw behavioral data are available on the Open Science Framework (T-maps: https://osf.io/9uzm8/; Stan model code: Base-Model: osf.io/6uz8g; Offset-Model: osf.io/mgjx5; Raw data: https://osf.io/nxcas).

## Author Contributions

Kilian Knauth: Conceptualization, Methodology, Software, Validation, Formal analysis, Investigation, Data Curation, Writing—Original Draft, Writing—Review & Editing, and Visualization. David Mathar: Conceptualization, Methodology, Software, Validation, and Writing—Review & Editing. Bojana Kuzmanovic: Software, Resources, and Writing—Review & Editing. Marc Tittgemeyer: Resources, Writing—Review & Editing. Jan Peters: Conceptualization, Methodology, Validation, Resources, Writing—Review & Editing, and Supervision.

## Declaration of Competing Interest

The authors declare no competing interests.

## Supplementary Material

Supplementary Material
