# Supplementary material for: Erotic cue exposure increases neural reward responses without modulating temporal discounting
Source: Imaging Neurosci (Camb). 2023 Aug 18;1:imag-1-00008. doi: 10.1162/imag_a_00008 (PMC12007530; doi:10.1162/imag_a_00008)
Supplement: Supplementary Material [file imag_a_00008-supp.pdf]

Supplementary Information

Supplementary Table S1. Peak voxels and SVC corrected *p*-values for condition-wise SV-coding (erotic, neutral)

**Exp. Condition**	Area	Peak Voxel	z-value	p_{SVC}
Neutral	VMPFC	-8/54/-8	5.35	**<0.001***
	Striatum	-8/14/-6	3.61	0.075
	PCC	-8/-32/40	3.27	0.196
Erotic	VMPFC	-6/44/-4	5.13	**<0.001***
	Striatum	-8/10/6	3.96	**0.023***
	PCC	-8/-34/38	3.84	**0.035***

Note: Asterisks denote significant effects after small volume correction (SVC); VMPFC = ventromedial prefrontal cortex; PCC = posterior cingulate cortex.

Supplementary Table S2. Peak voxels and SVC corrected *p*-values for condition-wise LL-choice coding (erotic, neutral)

**Exp. Condition**	Area	Peak Voxel	z-value	p_{SVC}
Neutral	left DLPFC	-44/44/6	3.98	**0.009***
Erotic	left DLPFC	-40/46/2	3.68	**0.025***

Note: Asterisks denote significant activations after small volume correction (SVC); DLPFC = dorsolateral prefrontal

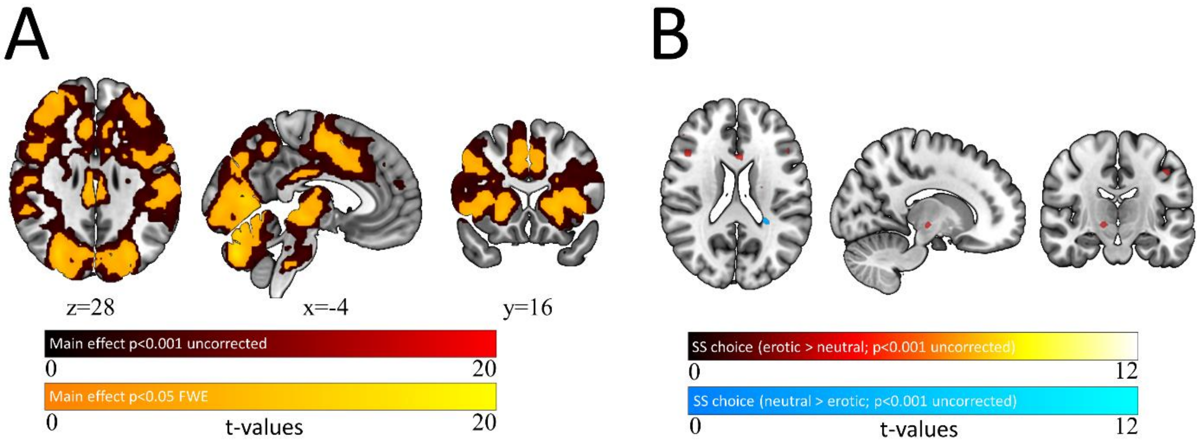

Supplementary Figure S1. Neuronal correlates of smaller-sooner choices. **A**: Display of the parametric (SS-) choice-regressor (mean across conditions); red, $p<0.001$ (uncorrected); yellow, whole-brain FWE corrected $p < 0.05$; **B**: Condition contrasts, erotic > neutral (red/yellow) and neutral > erotic (light blue), p<0.001 uncorrected (whole brain analysis).

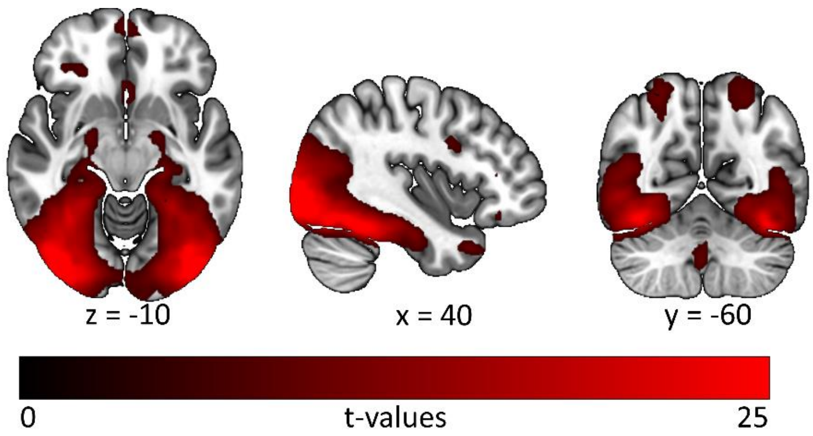

Supplementary Figure S2. Neuronal correlates intact vs. scrambled image processing irrespective of experimental condition. Red, whole-brain FWE corrected $p < 0.05$ (whole brain analysis).

Supplemental Table S3. Proportions of correctly predicted binary choices (mean [CIs]) for both included TD models (Base-Model, Offset-Model) split by experimental condition (see also Supplementary Figure S3).

Model	Neutral	Erotic
Base-Model	0.799 [0.762-0.836]	0.793 [0.753-0.833]
Offset-Model	0.831 [0.791-0.870]	0.812 [0.766-0.858]

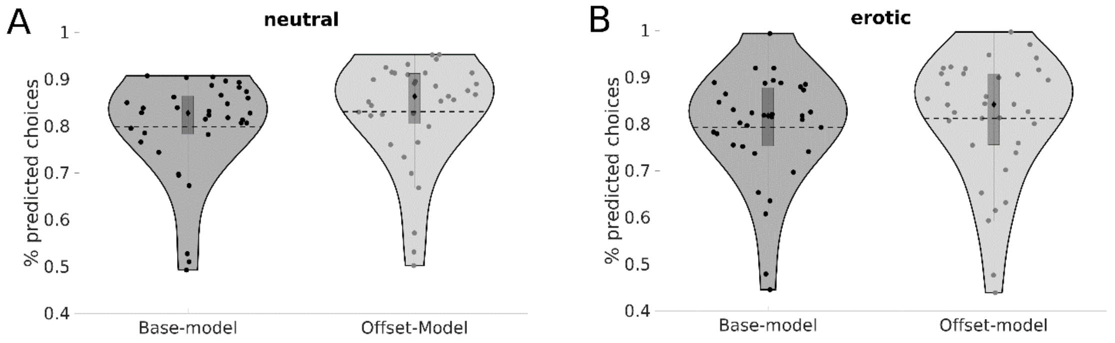

Supplementary Figure S3. Proportions of correctly predicted binary choices for the Base-Model and the Offset-model (including an SV-offset parameter ω) split by experimental condition (A: neutral, B: erotic); Dots = single subjects; Dashed lines = group means; Black diamonds = group medians.

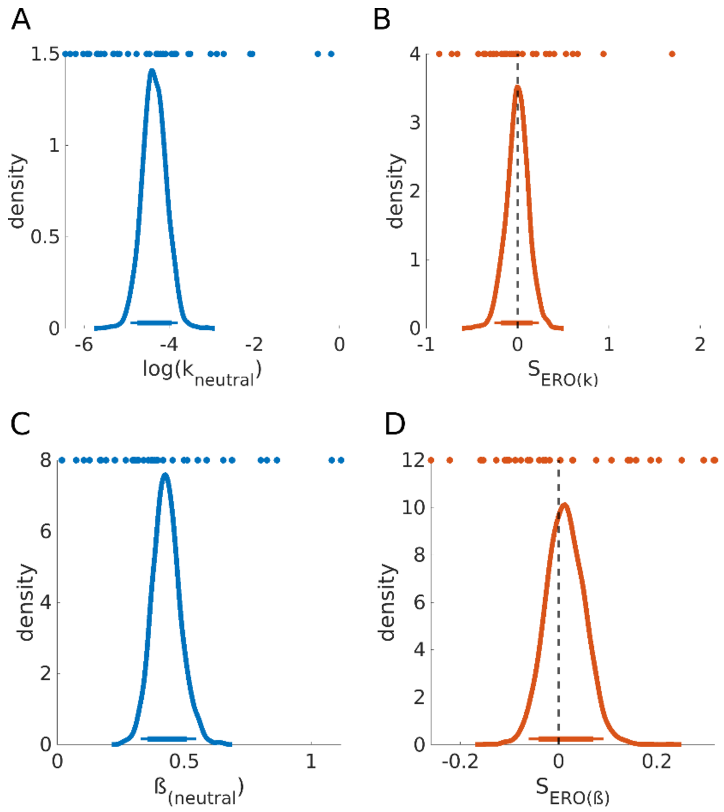

Supplementary Figure S4. Posterior distributions for log($k_{neutral}$) (A), $ß_{(neutral)}$ (C) and associated erotic shift parameters (B & D; Base-Model); Colored dots depict single subject means. Thick and thin horizontal lines indicate 85% and 95% highest density intervals.

Supplementary Table S4. Summary statistics of the posterior distributions of computational shift-parameters (Base-Model)

Parameter	Mean	SD	dBF	BF_{01}
SEro(k)	-0.004	0.484	1.030	5.810
SEro(ß)	0.014	0.154	0.530	30.807

Note. Abbreviations: BF_{01}, undirected Bayes factor in favor of null model; dBF, directional Bayes factor; *SD*, standard deviation.

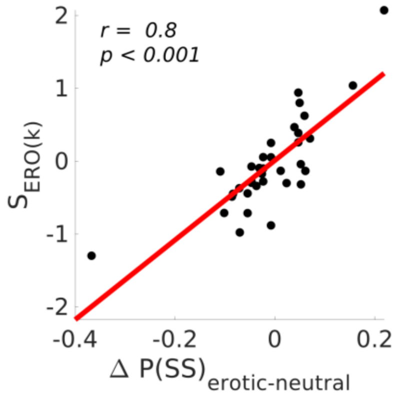

Supplementary Figure S5. Associations between model-free (SS-choice proportions) and model-based measures ($S_{Ero(k)}$) of temporal discounting behavior (Offset-model); *r* = Pearson's correlation coefficient.

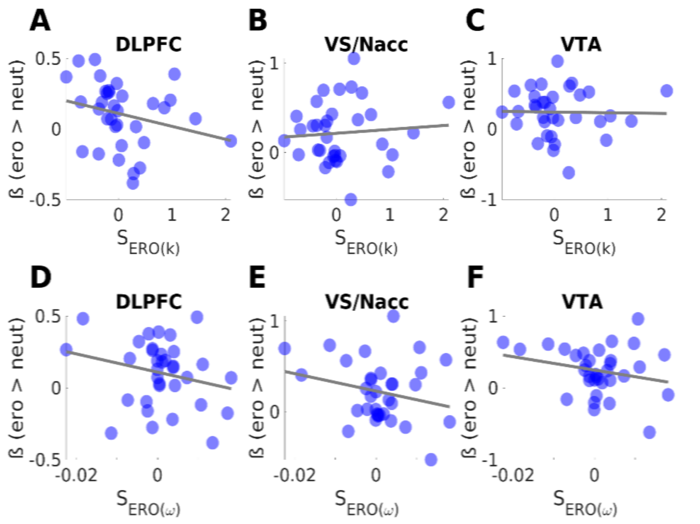

Figure S6. Associations between neuronal cue-reactivity-responses within key dopaminergic (VS/Nacc, VTA) and prefrontal (DLPFC) areas and subject-specific shift-parameters ($S_{Ero(k)}$, $S_{Ero(\omega)}$). Neuronal cue-reactivity within ROIs was quantified by extracting peak-voxel activity.

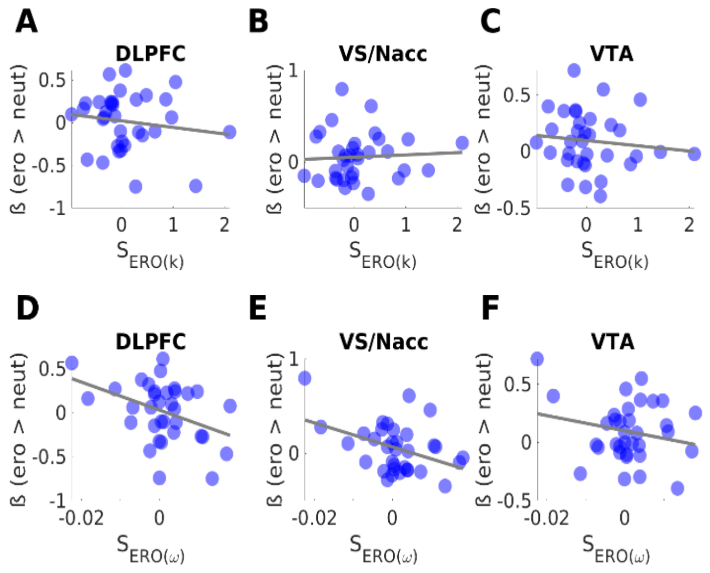

Figure S7. Associations between neuronal cue-reactivity-responses within key dopaminergic (VS/Nacc, VTA) and prefrontal (DLPFC) areas and subject-specific shift-parameters ($S_{Ero(k)}$, $S_{Ero(\omega)}$). Neuronal cue-reactivity within ROIs was quantified by extracting average voxel activity across the ROI.
